# Characterization of a lipid droplet and endoplasmic reticulum stress related gene risk signature to evaluate the clinical and biological value in hepatocellular carcinoma

**DOI:** 10.1186/s12944-022-01759-y

**Published:** 2022-12-29

**Authors:** Ziwei Guo, Jun Liang

**Affiliations:** 1 Peking University International Hospital, Beijing, China; 2 Peking University Cancer Hospital and Institute, Beijing, China

**Keywords:** Hepatocellular carcinoma, Gene risk signatures, Lipid droplet-associated protein, Endoplasmic reticulum stress, Overall survival, Immune microenvironment

## Abstract

**Introduction:**

Lipid metabolism and endoplasmic reticulum (ER) stress play an important role in the progression and metastasis of hepatocellular carcinoma (HCC). We aimed to establish lipid droplet (LD)-associated and ER stress-related gene risk signature as prognostic indicators.

**Materials and methods:**

Literature searches for LD-associated proteins was screened and validated in The Cancer Genome Atlas (TCGA) and International Cancer Genome Collaboratory (ICGC) databases. A total of 371 samples were enrolled from the TCGA RNA-seq dataset (training cohort) and 240 samples from IGGC RNA-seq dataset (validation cohort). A 10-gene risk signature was established by the last absolute shrinkage and selection operator (LASSO) regression analysis. The prognostic value of the risk signature was evaluated by Cox regression, Kaplan–Meier and ROC Curve analyses. Biological features associated with LD and ER stress-related factors were explored by functional analysis and in vitro experiment.

**Results:**

Based on the medical literatures, 124 lipid droplet-associated proteins were retrieved, and three genes failed to establish a valid prognostic model. ER stress was considered as an important component by functional analysis. A 10-gene risk signature compared the clinicopathology characteristics, immunosuppressive events and a nomogram in HCC patients.

**Conclusion:**

LD-associated and ER stress-related gene risk signatures highlighted poor prognosis for clinicopathological features, positively correlate with macrophages and T cell immunoglobulin and mucin-3 (TIM-3) expression in the tumor microenvironment, and might act as independent prognostic factors.

**Supplementary Information:**

The online version contains supplementary material available at 10.1186/s12944-022-01759-y.

## Introduction

HCC is the fourth highest cancer-related mortality worldwide [1, 2] and the second leading cause of cancer mortality [3]. As we known, hepatitis B virus (HBV) infection is a key undesirable factor but does not accurately predict the risk of causing HCC [3]. Contemporary epidemiological observations suggest that the etiology of cirrhosis and HCC is gradually changing from viral hepatitis to non-alcoholic fatty liver Disease (NAFLD)/ non-alcoholic Steatohepatitis (NASH) with the increase of metabolic diseases such as overweight and diabetes [4, 5]. Thus, viral pathogenesis and metabolic disease are jointly involved in the transition from NAFLD/NASH to HCC, a process that may be related to tissue lipid metabolism at least [6]. The presence of abnormal LD metabolism contributes to the development of HCC due to the involvement of the microbiota, insulin resistance, inflammation, and important cellular physiological processes, including cell division, expansion, differentiation, and motility [7, 8].

In addition, ER stress arises in the folding of proteins within the secretion pathway [9]. In a very recent study, an ER stress has been determined to be an important factor in HCC promoted by NAFLD [10] and involved in the promotion and advancement in many cancers [11, 12], as well as in the growth of cancer proliferation and resistance to radiation or chemotherapy in a hypoxic environment [13]. ER stress may be a worthy therapeutic candidate. Currently, the relationship between ER stress and the biologic features and prognosis of HCC is not yet clear. In addition, molecular characterization still does not predict prognosis, surgical success or risk of recurrence after ablation [14] or optimal treatment options [15]. It is becoming increasingly important whether the characterization of molecular mechanisms can guide clinicians in the comprehensive assessment of survival and biological features.

In this paper, no valid prognostic model was found when the differential genes after literature search were analyzed based on TCGA and ICGC datasets for LD-associated genes. In contrast, functional enrichment analysis by CRISPR/cas9 knockout library screening of Hep3B and SNU398 identified ER stress as an important factor involved. Therefore, a genetic risk signature was established by cross-genes of LD-associated and ER stress. This study explored clinicopathological features, immunological events and nomograms, which were found to be prominent in estimating 1, 3, and 5-year survivals in patients with HCC.

## Materials and methods

### Literature search for selecting LD-associated factors

The literature was searched in the PubMed database with the search formula "((LD Proteins or) AND Proteins, LD or) AND LD Coat Proteins" (last search date: February 22, 2022). There was no restriction on when the article was published, and the original publication date was detected as 1983. The categories "full text" and "free full text" were selected, excluding the categories "Review" and "Systematic review".

### Data collection

LD-associated genes and ER stress genes from Genecards (https://www.genecards.org/) and select these genes with correlation scores ≥ 7 as screening criteria. Tumor RNA-seq material (level 3) and medical data were obtained from TCGA (https://portal.gdc.com) and ICGC (https://dcc.icgc.org/releases/current/Projects) datasets.

### UALCAN analysis

Differentially expressed genes (DEGs), protein and survival analyses in the Clinical Proteomics Tumor Analysis Consortium (CPTAC) were undertaken in the UALCAN platform (http://ualcan.path.uab.edu/) [16, 17]. Then, univariate Cox regression and Kaplan–Meier analysis were utilized to determine LD-associated genes.

### Assistant for Clinical Bioinformatics (ACLBi) analysis

DEGs and overall survival (OS) analyses were conducted on the ICGC dataset (RIKEN, Japan) using the *ACLBi* (http://www.acbi.com) platform [18–22]. Box-line plots, univariate Cox regression, and Kaplan–Meier analysis were applied to identify LD-associated genes.

### Survival Analysis

Crossover genes were selected in TCGA and ICGC datasets, OS was compared across groups using the *survival* and *survminer* R packages, and time-dependent ROC curves predicting prognosis of HCC patients were created using the *survROC* R package to create time-dependent ROC curves in predicting the prognosis of HCC patients.

### Functional enrichment analysis

Gene Ontology (GO) and Kyoto Encyclopedia of Genes and Genomes (KEGG) pathway analyses were obtained from the Database for Annotation, Visualization and Integrated Discovery (DAVID) [23] (http://david.ncifcrf.gov/) to identify biological processes that are closely associated with risk signature. Further analysis of GO and KEGG pathways for risk scoring was performed using the following R software *clusterProfiler* package for differentially expressed genes.

### Construction and validation of LD and ER stress-related signature

The genes were narrowed down by performing LASSO regression by using the *glmnet* R package of the TCGA database. The risk score of each sample was determined using the formulas below:

$${\sum }_{\mathrm{i}=1}^{\mathrm{N}}\mathrm{\beta iXi}$$

In this equation, where βi is the value of expression of the LD-associated genes with χi being the Cox regression coefficients calculated by multivariate regression. Subsequently, the Akaike information criterion (AIC) method was used to perform the optimal survival risk model for LD-related genes and ER stress genes upon a linear integrated multivariate derived regression coefficients.

### cBioportal analysis of genetic alterations

The cBioportal (https://www.cbioportal.org/) [24, 25] was chosen to explore the list of 8 studies containing genetic alterations characteristic of HCC.

### The Human Protein Atlas (HPA) protein expression

The HPA website (https://www.proteinatlas.org/). was used to demonstrate protein expression for each of the 10 genes and to use the "Pathology" section to show the impact of protein levels on survival in HCC patients.

### Clinicopathological features

To determine whether risk features were correlated with clinicopathologic factors (including age, sex, and race, histological grade, T and TNM stage), correlations among these factors were presented as box plots.

### Immune cell fractions and immune checkpoints

Myeloid-derived suppressor cells (MDSCs) [26], Regulatory T (Treg) cells [26], natural killer (NK) T cells [27] and neutrophils [28] within the HCC micromanage were thought in association with immune suppression. Considering the key role played by tumor mutation burden (TMB) and microsatellite instability (MSI) in tumor immunosuppression and immunotherapy, correlations among risk characteristics and immune infiltrating cells, immune checkpoints, TMB and MSI were analyzed.

### Development and evaluation of the nomogram

Age, sex, race, viral infection, tumor grade, TNM and T stage, and risk score were combined to create the nomograms using the *survivals* and *rms* R packages. Calibration curves were used to assess the accuracy of nomograms in predicting 1-, 3-, and 5-year survival in patients with HCC [29, 30].

### Statistical analysis

This study mainly used R software (version 3.6.1) and GraphPad Prism v7.00 (GraphPad Software, Inc.). Wilcoxon test was used in comparing the variances among both groups, and the Kruskal- Wallis H test was applied for comparing multiple for multiple groups. Two-sided Mann–Whitney U test was performed for two-way comparisons in clinicopathological characteristics, and two-sided chi-square test or exact test for 2-independent samples was used for categorical variables. Quantitative data were calculated as the mean values with standard deviation (SD). *P* < 0.05 it was considered to be statistical significances.

## Results

### Selection of LD-associated factors

Based on the literature search and analysis, 124 results were found for LD- associated factors (Table S1, Fig. S1). The selection criteria included not only LD factors but also some protein-mediated LD related organelles, such as ER, mitochondria, Golgi apparatus and lysosomes.

### Differences of gene expression and OS are significant based on the TCGA dataset

RNA-seq expression data of 124 genes related to lipid metabolism based on the TCGA dataset were analyzed. A genetic heat map was drawn between HCC patient specimens and normal tissues (Fig. S2). Ninety-six DEGs were identified, including 71 up-regulated and 25 down-regulated genes (Table 1). Fourteen of these genes were selected due to differences in gene expression, OS, and univariate COX analysis (Figs. S3, S4, S5, S8a).

**Table 1:** The differences in gene expression, protein expression, and survival analysis of 125 genes in TCGA dataset

Factors	DEGs	OS	Protein expression	Factors	DEGs	OS	Protein expression	Factors	DEGs	OS	Protein expression
ABHD5	*	/	/	FGF21	****↑	*	/	PLIN3	****↑	***	****↓
ACAT1	****↓	*	/	FIG4	****↑	****	****↓	PLIN4	**↓	*	/
ACSL3	****↑	***	****↑	FITM1	****↓	*	/	PLIN5	****↑	*	/
ACSL4	****↑	*	/	FITM2	****↑	**	*	PNPLA2	****↑	*	/
ACOX1	****↓	*	/	G0S2	**↓	*	/	PNPLA3	**↓	*	/
AGPAT2	**↓	*	/	GAPDH	****↑	***	****↓	PNPLA4	*	/	/
AIFM2	****↑	**	**↑	GBF1	****↑	*	/	PNPLA5	*	/	/
ANXA2	****↑	**	****↑	GIMAP2	****↑	*	/	PRPF19	****↑	****	****↑
APOA4	***↓	*	/	HSD17B11	***↑	*	/	RAB18	****↑	**	*
APOB	****↓	*	/	HSD17B13	****↓	*	/	RAB3GAP1	****↑	*	/
AQP1	**↑	*	/	HSD3B7	****↑	*	/	RAB5A	****↑	*	/
ARAP2	*	/	/	HSPA5	****↑	**	*	RAB5C	****↑	**	*
ATG2A	*	/	/	IRAK1	****↑	**	****↑	RAB7A	****↑	****	****↓
ATG2B	***↓	*	/	LIPE	***↑	*	/	RAB8A	****↑	*	/
AUP1	****↑	****	****↑	LMLN	****↑	*	/	RAP1B	****↑	**	****↓
BCAP31	****↑	*	/	LPCAT1	****↑	****	****↑	RBP1	***↓	*	/
BSCL2	****↑	*	/	LPCAT2	****↑	**	*	RSAD2	*	/	/
CAV1	****↑	*	/	LPIN1	****↑	*	/	SCCPDH	****↑	*	/
CAV2	****↓	*	/	LSS	****↑	*	/	SCD	****↑	*	/
CDKN1A	*	/	/	MAP4K4	****↑	*	/	SET	****↑	****	****↑
CES1	***↓	*	/	MBOAT7	****↑	****	****↑	SIGMAR1	****↑	*	/
CIDEA	*	/	/	METTL7A	****↓	*	/	SNAP23	****↑	*	/
CIDEB	****↓	*	/	METTL7B	*	/	/	SPAST	****↑	**	***↑
CIDEC	****↑	**	-	MGLL	*	/	/	SPG20	****↓	**	-
CKAP4	****↑	***	****↑	MTTP	****↓	*	/	SQLE	****↑	**	****↑
CYB5R3	****↑	***	****↓	NAPA	****↑	*	/	STARD13	****↑	*	/
DBC1	*	/	/	NCEH1	****↑	*	/	STX5	****↑	*	/
DFFA	****↑	***	*	NNMT	****↓	*	/	SYNGR2	****↑	**	****↑
DGAT1	****↑	*	/	NSDHL	****↑	*	/	TMEM135	****↑	*	/
DGAT2	***↓	*	/	NSF	****↑	*	/	TRAF6	****↓	*	/
EDA	****↑	**	-	OSBPL2	****↑	*	/	TSC1	****↑	***	*
EHD1	****↑	****	****↓	PCYT1A	****↑	***	****↑	UBE2G2	****↑	*	/
FAAH2	*	/	/	PEMT	****↓	*	/	VAMP4	****↑	*	/
FABP1	****↓	*	/	PITPNM1	****↑	*	/	VAPA	****↑	*	/
FABP4	****↑	*	/	PLD1	*	/	/	VCP	****↑	*	/
FAF2	****↑	***	****↑	PLIN1	***↓	*	/				
FASN	****↑	*	/	PLIN2	****↓	*	/				

*TCGA* The Cancer Genome Atlas, *DEGs* Differentially expressed genes, *OS* Overall survival

^****^ *P* < 0.001

^***^ *P* = 0.01–0.001

^**^ *P* = 0.05–0.01

^*^ *P* ≥ 0.05

Six genes (annexin A2 (ANXA2), b cell receptor-associated protein 31 (BCAP31), cytoskeleton-associated protein 4 (CKAP4), hydroxysteroid 13 (HSD17B13), interleukin-1 receptor-associated kinase (IRAK1), squalene epoxidase (SQLE)) was screened by the ICGC database (Table 2). ANXA2 is correlated with therapeutic resistance to various cancer forms [31]; BCAP31 encoded protein involved to transport membrane proteins from ER to Golgi [32]; CKAP4 is an obsolete signal transducer activity and involved in protein metabolism [33]; HSD17B13 has steroid dehydrogenase activity and functions within the upstream or positive regulation of lipid biosynthetic progress [34, 35]; IRAK1 provides protein kinase activity and contributes to the partial upregulation of the IL 1-induced transcription factor NF-kappa B [36]; SQLE acquires oxidoreductase activity, the first oxygenative step in sterol biosynthesis [37]. In terms of ER activity and protein-lipid metabolism, these six genes play different roles in regulating cell growth and signal transduction pathways.

**Table 2:** The differences in gene expression, Kaplan–Meier survival analysis, and COX analysis of 114 genes in ICGC dataset

Factors	DEGs	Log rank *P*	Cox *P*	Factors	DEGs	Log rank *P*	Cox *P*	Factors	DEGs	Log rank *P*	Cox *P*
ABHD5	*	/	/	FIG4	*	/	/	PLIN2	*	/	/
ACAT1	*	/	/	FITM1	****↓	*	/*	PLIN3	*	/	/
ACSL3	*	/	/	FITM2	*	/	/	PLIN4	*	/	/
ACSL4	****↑	*	*	G0S2	****↓	*	/	PLIN5	*	/	/
AGPAT2	*	/	/	GAPDH	*	/	/	PNPLA2	*	/	/
AIFM2	*	/	/	GBF1	*	/	/	PNPLA3	*	/	/
ANXA2	****↑	***	**	GIMAP2	*	/	/	PNPLA4	*	/	/
APOA4	****↓	*	/	GPAT4	*	/	/	PRPF19	*	/	/
APOB	*	/	/	HILPDA	*	/	/	RAB18	*	/	/
AQP1	*	/	/	HSD17B11	*	/	/	RAB3GAP1	*	/	/
ARAP2	*	/	/	HSD17B13	****↓	***	***	RAB5A	*	/	/
ATG2A	*	/	/	HSD3B7	*	/	/	RAB5C	*	/	/
ATG2B	*	/	/	HSPA5	*	/	/	RAB7A	*	/	/
AUP1	*	/	/	ICE2	*	/	/	RAB8A	*	/	/
BCAP31	****↑	***	***	IRAK1	****↑	****	***	RAP1B	*	/	/
BSCL2	*	/	/	LDAH	*	/	/	RBP1	****↓	*	/
CAV1	*	/	/	LIPE	*	/	/	RSAD2	*	/	/
CAV2	*	/	/	LMLN	*	/	/	SCCPDH	*	/	/
CAVIN1	*	/	/	LPCAT1	*	/	/	SCD	*	/	/
CDKN1A	*	/	/	LPCAT2	*	/	/	SET	*	/	/
CES1	*	/	/	LPIN1	*	/	/	SIGMAR1	*	/	/
CIDEB	****↓	*	/	LSS	*	/	/	SNAP23	*	/	/
CIDEC	*	/	/	MAP4K4	*	/	/	SPAST	*	/	/
CKAP4	****↑	***	****	MBOAT7	*	/	/	SQLE	****↑	***	**
CPT1A	*	/	/	METTL7A	*	/	/	STARD13	*	/	/
CTDNEP1	*	/	/	METTL7B	*	/	/	STX5	*	/	/
CYB5R3	*	/	/	MGLL	*	/	/	SYNGR2	*	/	/
DFFA	*	/	/	MTTP	*	/	/	TMEM135	*	/	/
DGAT1	*	/	/	NAPA	*	/	/	TPD52	*	/	/
DGAT2	*	/	/	NCEH1	*	/	/	TRAF6	*	/	/
DNAAF1	*	/	/	NNMT	****↓	*	/	TSC1	*	/	/
EDA	*	/	/	NSDHL	*	/	/	UBE2G2	*	/	/
EHD1	*	/	/	NSF	*	/	/	VAMP4	*	/	/
FAAH2	*	/	/	OSBPL2	*	/	/	VAPA	*	/	/
FABP1	****↓	*	/	PCYT1A	*	/	/	VCP	*	/	/
FABP4	*	/	/	PEMT	****↓	*	/	VMP1	*	/	/
FAF2	*	/	/	PITPNM1	*	/	/				
FASN	*	/	/	PLD1	*	/	/				
FGF21	*	/	/	PLIN1	*	/	/				

*ICGC* International Cancer Genome Collaboratory, *DEGs* Differentially expressed genes

^****^ *P* < 0.001

^***^ *P* = 0.01–0.001

^**^ *P* = 0.05–0.01

^*^ *P* ≥ 0.05

In HCC cells, these genes were upregulated except for HSD17B13 (Fig. S6). The high expression of this gene indicated a good prognosis of patients (Fig. S7) and was considered as a protective factor (Fig. S8b). Based on the available literature, six genes were identified, but similar results could not be obtained in both databases by further prognostic analysis. Finding intrinsic association mechanisms to optimize risk models for genes becomes critical.

### Valueless prognostic model in TCGA and ICGC database and biological function in HCC

Three genes (ANXA2, CKAP4, and IRAK1) from the TCGA and ICGC datasets were screened. The higher level of these genes presented the poorer prognosis, and the AUC of these genes predicting OS decreased gradually over time in both datasets (Fig. S9a, b, S10a, b, c, d). However, these genes did not serve as valid factors for the nomogram to predict the 1-, 3-, 5-year survival probabilities of patients in the TCGA dataset (Fig. S11a, b, c, d). In other words, the high expression of the three genes in the TCGA and ICGC databases represented a poor prognosis, but did not establish a valid prognostic model. Therefore, these three genes do not serve as valuable markers to improve clinical guidance.

**Fig. 1:**
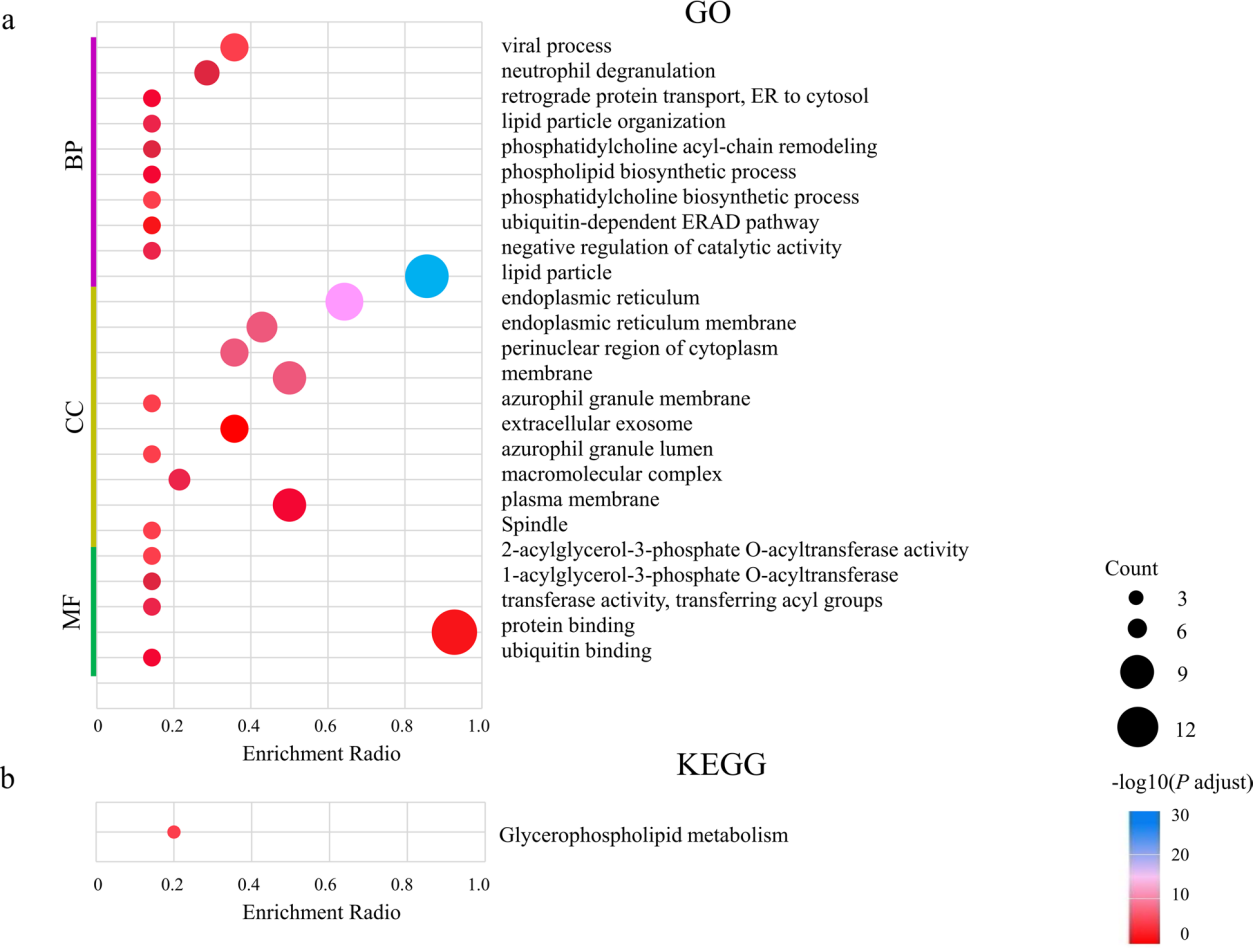
Functional enrichment analysis based on differentially expressed genes from literature research. **a** GO analysis. **b** KEGG pathway analysis. GO: Gene Ontology, KEGG: Kyoto Encyclopedia of Genes and Genomes, BP: biological processes, CC: cellular components, MF: molecular function

To explore more information about lipid related information in HCC, GO and KEGG pathway analysis was performed by DAVID online tool based on the list of genes provided above. As shown in Fig. 1, the functions of these genes were mainly focused on processes such as protein transport and lipid granulation from the ER to the cytoplasm (Fig. 1a); the KEGG pathway showed that this signal was positively correlated with the glycerophospholipid metabolic signaling pathway (Fig. 1b). However, KEGG and GO analyses were not performed for HCC. Therefore, the intrinsic association associated with HCC was verified and screened in the next cellular experiments performed.

### CRISPR/cas9 library screening identify ER stress as a strong correlate factor of LDs

**Fig. 2:**
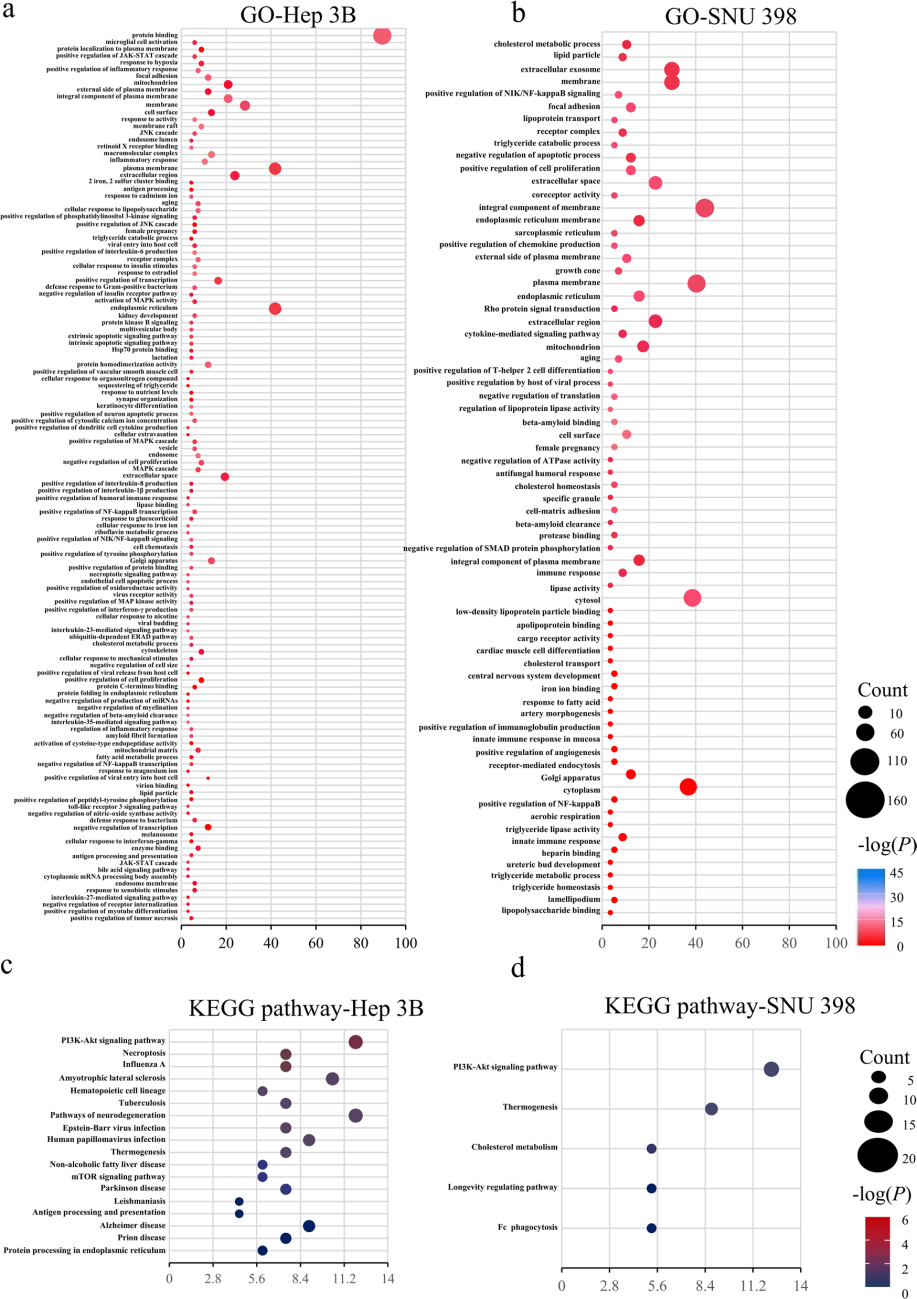
Functional enrichment analysis based on differentially expressed genes from CRISPR/cas9 knockout library screening of Hep 3B cells and SNU398 cells about lipid droplet-associated genes from Genecards tool. **a**, **b** GO analysis in Hep 3B and SNU 398 cells in the TCGA cohorts. **c**, **d** KEGG pathway analysis in Hep 3B and SNU 398 cells in the TCGA cohorts. GO: Gene Ontology, KEGG: Kyoto Encyclopedia of Genes and Genomes

The specific process of CRISPR/cas9 knockout library screening was reported in our previous study [38]. Sixty-seven LD-associated genes were found in Hep3B cells and 57 LD-associated genes were enrolled in SNU398 cells from the Genecards tool. Functional enrichment analysis revealed that in Hep3B cells, the top 5 positions of GO analysis were protein binding, plasma membrane, ER, mitochondria and active regulatory of RNA Polymerase II initiator transcription and membrane components, plasma membrane, cytoplasm, ER membrane and ER and mitochondria were revealed in SNU398 cells (Fig. 2a, b). The ER protein pathway and cholesterol metabolism were illustrated in KEGG pathway of the Hep3B cells and the SNU398 cells, separately (Fig. 2c, d). This part of the results is more indicative of the fact that the strongest intrinsic link to LDs correlation in HCC is ER stress, further validating the results obtained from online database. Screening for crossover genes for LDs and ER stress was next continued.

### Establishing and evaluating the risk signature of LD and ER stress- associated genes

**Fig. 3:**
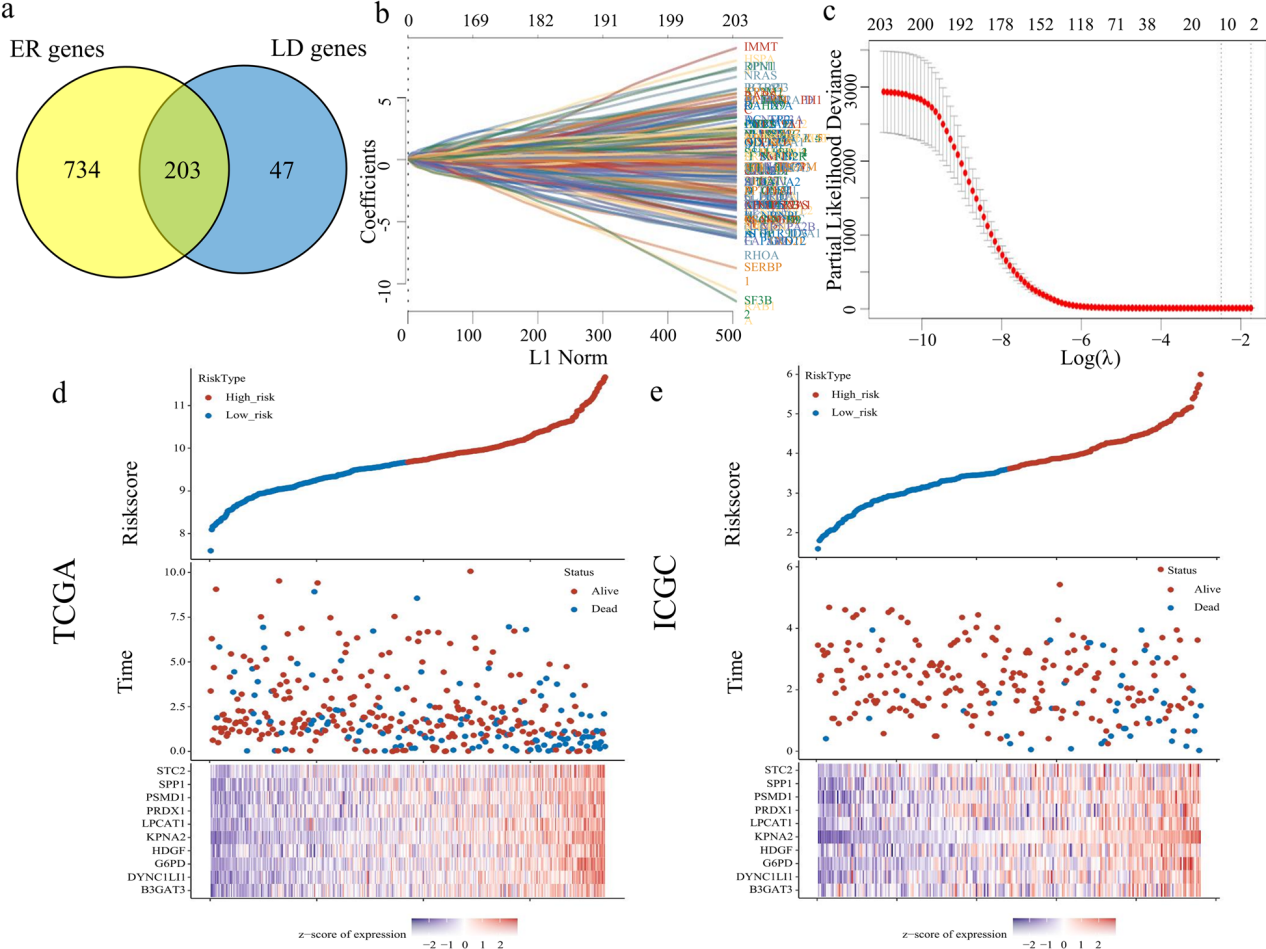
Identifying prognostic genes for developing a risk model. **a** Intersecting genes associated with HCC OS in the TCGA and ICGC datasets. **b** LASSO coefficient profiles of the 203 genes in the TCGA data set. **c** Selection of the optimal parameter (lambda) in the LASSO model. **d** The distribution of the risk score and survival overview about the ten genes chosen for establishing a prognosis signature in the TCGA cohort. **e** The distribution of the risk score and survival overview about the ten genes chosen for establishing a prognosis signature in the ICGC cohort. LASSO, least absolute shrinkage and selection operator; HCC hepatocellular carcinoma, OS, overall survival; TCGA, The Cancer Genome Atlas; ICGC, International Cancer Genome Collaboratory

**Fig. 4:**
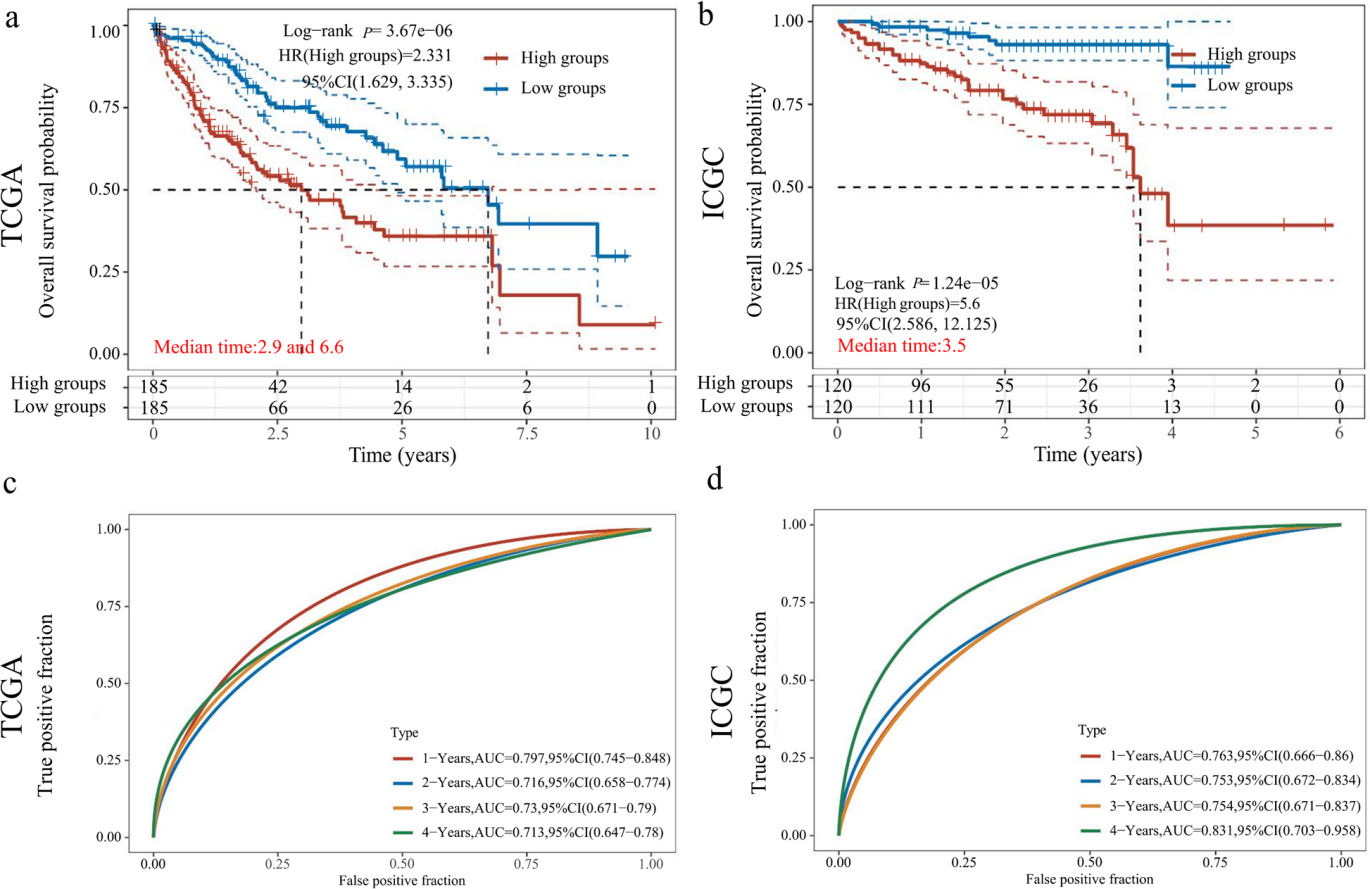
The prognostic value of the LD-associated and ER stress-related gene risk signature in TCGA and ICGC datasets. **a**, **b** K-M survival analyses of the risk signature in HCC patients. **c**, **d** The time ROC curve analyses were performed to predict 1-, 2-, 3-, and 4-year OS according to risk score in the TCGA and ICGC datasets. LD lipid droplet, ER endoplasmic reticulum, TCGA The Cancer Genome Atlas, ICGC International Cancer Genome Collaboratory

A total of 1959 LD-associated genes (search formula: LD-associated liver carcinoma) and 7235 ER stress genes (search formula: ER stress liver carcinoma) was selected in liver cancer patient specimens, and 203 crossover genes were performed by LASSO regression analysis (Fig. 3b, c) between 250 LD-associated genes and 937 ER stress genes from the TCGA database (Fig. 3a). Subsequently, 10 genes were identified, namely: stanniocalcin 2 (STC2), secreted phosphoprotein 1 (SPP1), proteasome 26S subunit, non-ATPase 1 (PSMD1), peroxiredoxin 1 (PRDX1), lysophosphatidylcholine acyltransferase 1 (LPCAT1), karyopherin subunit alpha 2 (KPNA2), heparin binding growth factor (HDGF), glucose-6-phosphate dehydrogenase (G6PD), dynein cytoplasmic 1 light intermediate chain 1 (DYNC1LI1), beta-1,3-glucuronyltransferase 3 (B3GAT3). Risk score (TCGA) = (0.3901)* B3GAT3 + (0.1809)* DYNC1LI1 + (-0.0227)* G6PD + (0.3367)* HDGF + (0.1425)* KPNA2 + (0.0557)* LPCAT1 + (0.1757)* PRDX1 + (0.161)* PSMD1 + (0.0263)* SPP1 + (0.0725)* STC2; AIC (ICGC) = 414.2736; risk score (ICGC) = (0.066)* B3GAT3 + (-0.7061)* DYNC1LI1 + ( 0.0469)* G6PD + (0.1462)* HDGF + (0.8041)* KPNA2 + (0.1403)* LPCAT1 + (-0.1)* PRDX1 + (0.2207)* PSMD1 + (0.0254)* SPP1 + (0.0245)* STC2 (Fig. 3d, e). The high-risk signatures of 10 genes in both datasets suggested poor prognosis (TCGA: HR 2.331, 95%CI 1.629–3.335, *P* = 3.67e-06; ICGC: HR 5.6, 95%CI 2.586–12.125, *P* = 1.24e-05) (Fig. 4a, b). The AUCs for 1-, 2-, 3-and 4-year OS in the TCGA dataset were 0.797, 0.716, 0.73 and 0.713, respectively. The AUCs for predicting 1-, 2-, 3-and 4-year OS in the ICGC data set were 0.763, 0.753, 0.754 and 0.831, respectively (Fig. 4c, d). These represented a decrease in the ability of the TCGA dataset to predict OS, but an increase in the ability of the ICGC dataset to predict OS. In addition to screening for valid genetic risk scores, the expression of these genes and their correlations were subsequently analyzed.

### Gene alteration and protein expression of the risk signature

The correlation of these 10 genes in the TCGA and ICGC datasets was analyzed (Fig. S12a, b). Among them, STC2 to be expressed in a diverse variety of tissues and related pathways are protein metabolism and regulation of insulin-like growth factor (IGF) transport [39]; SPP1 is the cytokine to upregulate the expression of interferon-gramma and interleukin-12, cytokine activity and extracellular matrix binding [40]; PSMD1 is proteasome that works in a non-lysosomal pathway in an ATP/ubiquitin-dependent process cleaves peptides [41]; PRDX1 encoded a protein that probably functions as an antioxidant protector in cells and might facilitate the anti-viral activity of CD8( +) T cells [42]; LPCAT1 encoded an enzyme that acts in the metabolism of phospholipids, in particular the transformation of lysophophatidylcholine into phosphatidylcholine when acyl-CoA is present [43]; PNA2 protein interacts with nuclear localization sequence (NLSs) of DNA helicases and may participate in protein nuclear transport [44]; HDGF encoded a protein with mitogenic and DNA-binding activities that possibly serves a need for cell proliferation and differentiation [45]; The main function of G6PD is the production of NADPH, which is a defense against oxidants and reductive biosynthetic reactions in key electron donor [46]; BYNC1LI1 encoded a protein that is involvement in intrastromal transport and chromosomal separation in mitosis. This protein could potentially in turn intermediate association with additional cargo molecules in order to promote transport of intracellular vesicles. [47]; B3GAT3 catalyzes the formation of the glycosaminoglycan-protein linkages through glucuronide transfer reactions in the final step of the linkage region biosynthesis of proteoglycans [48]. Together, these 10 genes are involved in the progression of RNA binding, transport and metabolism related to three substances (proteins, lipids and glucose) and cell proliferation. The roles of these genes are consistent with lipid metabolism and ER stress-related functions.

DYNC1LI1 and KPNA2 showed the highest correlation in both datasets (correlation coefficient: 0.7). In the online database, cBioportal, a total of 1571 patients/samples were found to have genetic alterations in HCC. Gene alterations were found in 250 (15.90%) of the interviewed patients/samples (Fig. S12c). HDGF (6%) had the highest frequency of genetic alterations. In addition, these 7 genes had high levels of protein expression and were risk factors that demonstrated a poorer prognosis in HCC patients (Fig. S13). A risk score for the 10 gene signature was subsequently screened and identified, which was further known to be associated with a poorer prognosis for HCC based on protein expression and correlation analysis. Therefore, does this risk score correlate to the clinicopathologic characteristics for HCC? This was analyzed below.

### Clinicopathological features

**Fig. 5:**
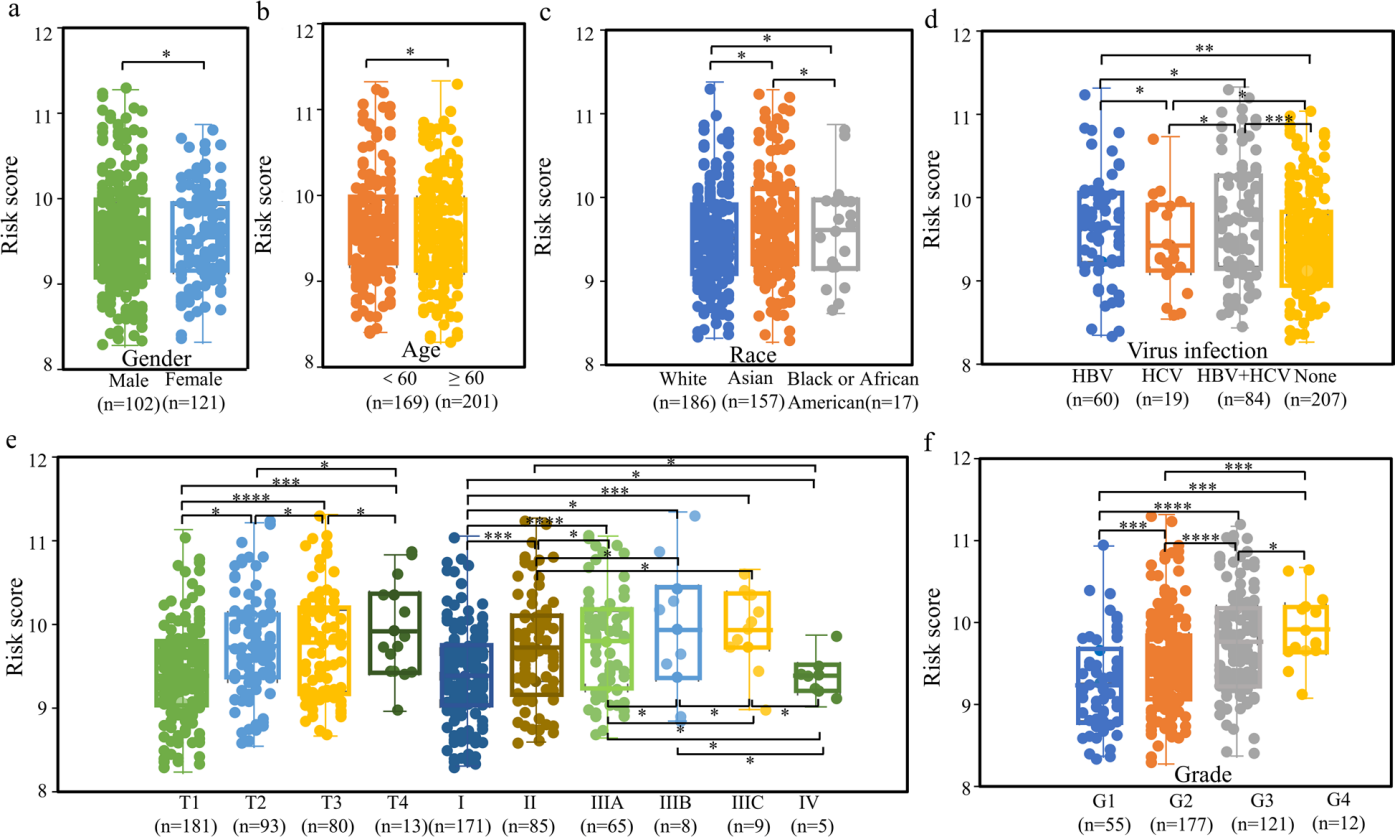
Association between the signature and clinicopathologic features in the TCGA datasets. The association between risk score and gender (**a**), age (**b**), race (**c**), virus infection (**d**), TNM and T stage (**e**) and grade (**f**) of HCC patients. **** *P* < 0.001, *** *P* < 0.01, ** *P* < 0.05, * *P* ≥ 0.05. TCGA The Cancer Genome Atlas, HCC hepatocellular carcinoma

Clinicopathological presentation according to the risk score, gender, age, race, HBV/HCV, T and TNM-stage, and histological grading from TCGA and ICGC datasets. There were no differences between risk scores and patients' age, gender, and race (Fig. 5a, b, c, Fig. S14a, b). In addition, risk scores were higher in patients with HBV/HBV + HCV infection than in patients without viral infection in the TCGA dataset (Fig. 5d).

Risk scores were higher in T-stage with deeper local infiltration, especially with significant differences among early (T1, I) and advance (T3, T4, II, IIIA, IIIC) stage. The stage IV had the lowest score, but was not different from the early stage (Fig. 5e). Unlike the results of the TCGA dataset, risk scores were not well differentiated among T-stage and stages III and IV. However, early (I and II) and advanced (III and IV) stages could be more clearly distinguished (Fig. S14c). In the TCGA dataset, this risk score could effectively distinguish difference among histological grades except for grades 3 and 4 (Fig. 5f, S14d). This result suggests that this risk score can effectively differentiate between early- and late- stage patients and there are significant differences in patients with chronic viral infections. As we know, the treatment of HCC is still focused on the combination of immune drugs, so is there a correlation between this risk score and immune checkpoints and immune cells to assist in clinical guidance?

### Immune checkpoints and immune cells

**Fig. 6:**
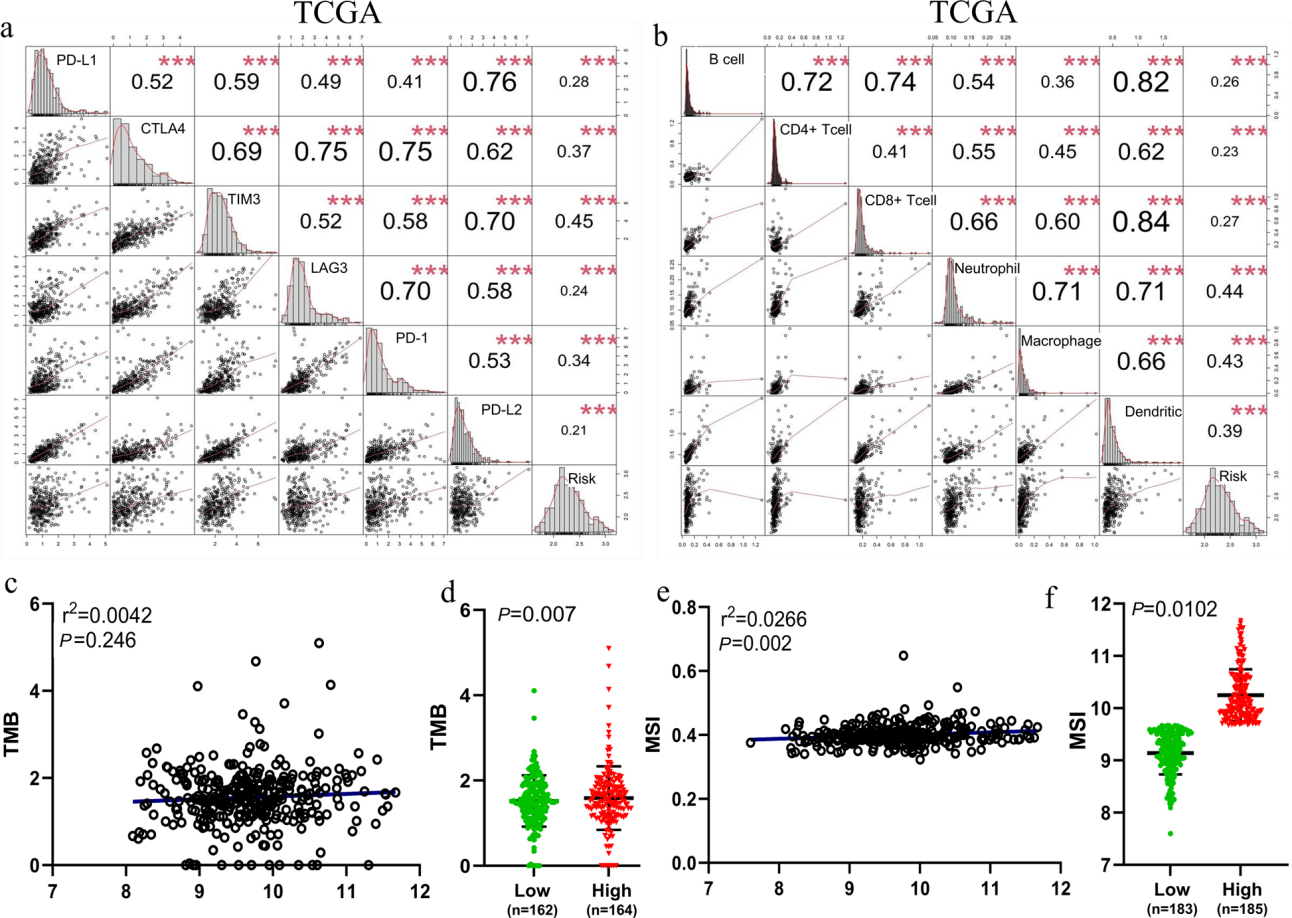
Relationship between the risk signature and immune checkpoints and immune cells in the TCGA cohort (Pearson correlation analysis). **a** Correlation between the risk scores and the expression of PD-L1, CTLA-4, TIM3, LAG-3, PD-1 and PD-L2. **b** Correlation between the risk scores and the expression of B cell, CD4 + T cell, CD8 + T cell, neutrophil, macrophage and dendritic cells. Correlation between the risk score and the expression of TMB in each patient (**c**) and low- and high- groups (**d**). Correlation between the risk score and the expression of MSI-high in each patient (**e**) and low- and high- groups (**f**). **** *P* < 0.001, *** *P* < 0.01, ** *P* < 0.05, * *P* ≥ 0.05. TCGA The Cancer Genome Atlas, PD-L1/2 programmed death ligands 1/2, CTLA-4 cytotoxic T lymphocyte-associated antigen 4, TIM-3 T cell immunoglobulin and mucin-3, LAG-3 lymphocyte-activation gene 3, PD-1 programmed death 1

Previous investigations reported that LDs has an important effect in the immune and inflammatory response in a variety of diseases [49]. Several immune checkpoint genes were included in this study, such as cytotoxic T lymphocyte-associated antigen 4 (CTLA-4), programmed death 1 (PD-1) and its ligands PD-L1 and PD-L2, TIM-3 and lymphocyte-activation gene 3 (LAG-3). Risk scores were positively correlated with most immune checkpoint genes and several immune cells, especially TIM-3 (Fig. 6a, S15a), neutrophils, and macrophages (Fig. 6b). This suggested that neutrophil-and macrophage-mediated immune or inflammatory responses are associated with samples from high-risk populations [50, 51]. Unfortunately, there was no difference in TMB expression levels between low- and high- risk scores (Fig. 6c), but there was a significant positive correlation between MSI and TMB expression levels in the high-risk population (Fig. 6d, e, f). TIM-3 expression was significantly increased in HCC tissues and promoted Treg cell proliferation and induced apoptosis of CD8 + T lymphocytes. In addition, TIM-3 expression was significantly increased on macrophages in HCC tissues, and its expression was positively correlated with HCC stage and negatively correlated with patient survival [52]. Thus, TIM-3 may downregulate the immune response of patients to HCC and promote the continued the development and spread of cancer cells. Meanwhile, patients with high genetic risk have more MSI and high TMB, which seems to contradict the status of the immune microenvironment. In the ICGC data set, the levels of TMB did not reach statistical differences either in distinguishing the high-risk and low-risk groups or in each patient (Fig. S15b, c). In both TCGA and ICGC databases, an analysis of the correlation of risk score with common immune checkpoints and immune cells told us that this score had strongest correlation with TIM3, neutrophils, and macrophages, while at low and high levels, the correlation of the risk score with TMB is inconsistent in both databases. Whether this risk score is meaningful for the prognostic model is discussed next.

### Construction and validation of the nomogram

**Fig. 7:**
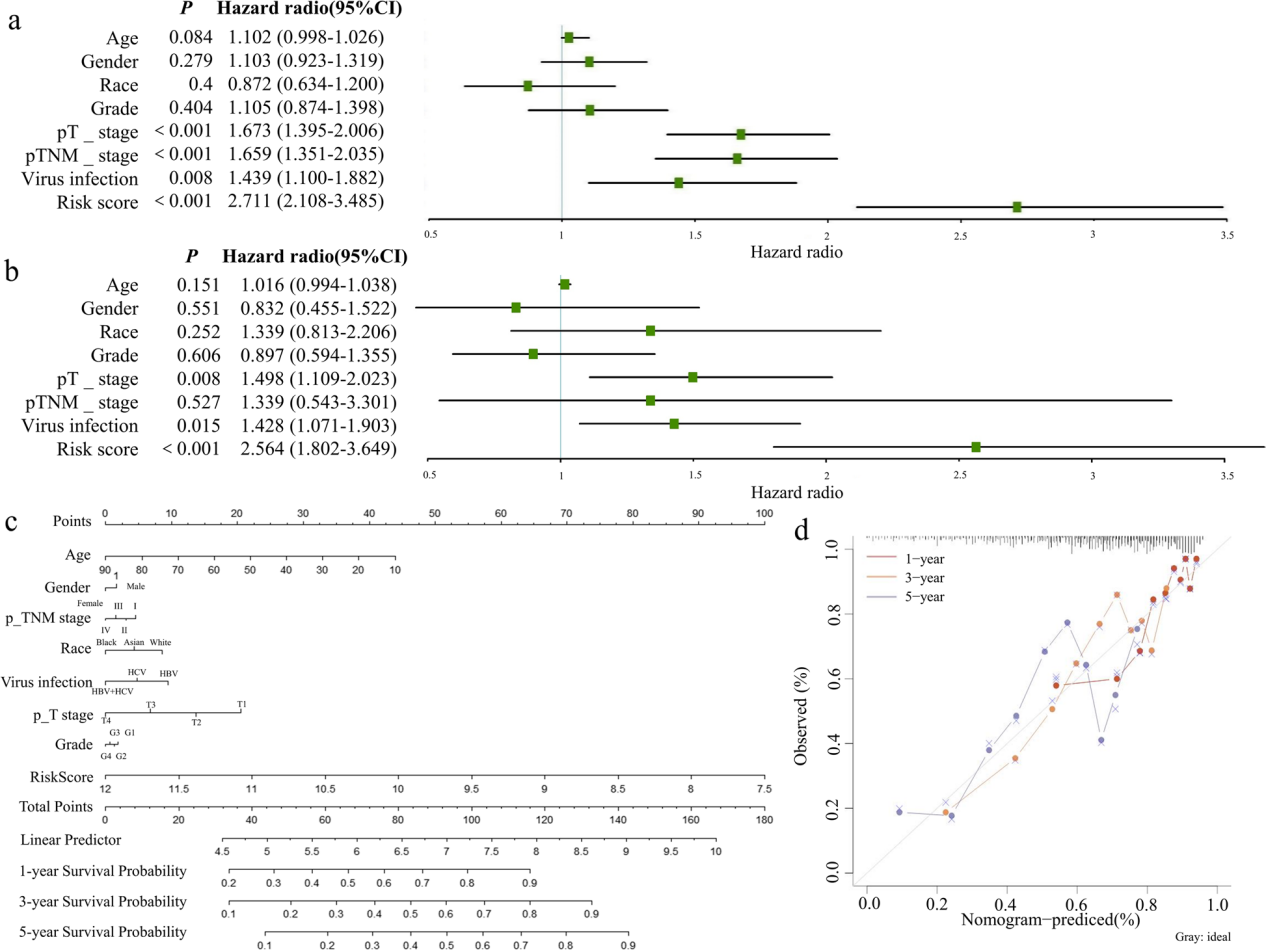
The prognostic value of the LD-associated and ER stress-related genetic risk signature in TCGA dataset. **a** Univariate and (**b**) multivariable analyses of 10 genes in the TCGA dataset. **c** The nomogram and (**d**) the calibration curve analyses were performed to predict 1-, 3-, and 5-year OS according to risk score. LD lipid droplet, ER endoplasmic reticulum, TCGA The Cancer Genome Atlas, OS overall survival

It is necessary to understand whether risk characteristics can serve as independent predictive factors for prognosis. In the TCGA dataset, univariate Cox analysis showed that OS in HCC patients showed a significant negative correlation with risk scores. Furthermore, T-stage, TNM-stage, and viral infection status suggested worse OS (Fig. 7a). Subsequent multifactorial Cox analysis showed that risk scores with T stage and viral infection could be independent prognostic factors (Fig. 7b).

In addition, the nomogram created integrated the above factors to predict OS at 1, 3, and 5 years (Fig. 7c). The calibrated curve indicated a notable consensus that real time to survival in the 1-year, 3-year, and 5-year TCGA cohort showed good agreement with the predicted survival rate although its ability to predict OS diminished over time (Fig. 7d). And similar results were validated in the ICGC dataset (Fig. S16a, b, c, d).

## Discussion

The etiopathogenesis and the progress in HCC is a multifactorial systematic and multistep process [53]. Due to the highly aggressive and heterogeneity of viral oncogenic cells, treatment strategies vary across geographic regions, mainly due to the lack of a relatively strong scientific basis [54–56]. In recent years, immune checkpoint inhibitors have been shown to be effective immune responses to eliminate tumor cells [57, 58]. In the near future, immune checkpoint-based therapies may also improve the efficacy of local and radical treatments for liver cancer as well as neoadjuvant therapy. In this process, researches to translate molecular and immune categories into biomarkers to guide therapy are still ongoing. Among them, more concrete progress has been made in understanding the underlying mechanisms of adiposity disease-associated HCC, with new insights into the role of the tumor microenvironment, particularly the immune system, in the pathophysiology of the disease [59]. Lipid aggregation is associated with ER stress, oxidative stress, mitochondrial defects, cholesterol efflux factors, ER autophagy, protein post-translational modifications and autophagy [60]. Among them, ER stress induces the release of pro-inflammatory factors from immune cells, while inhibiting the ability of antigen delivery. ER stress function is emphasized due to stress signals, such as inositol-requiring enzyme 1 alpha (IRE1α) and protein kinase RNA like endoplasmic reticulum kinase (PERK), leading to the suppression of immune cells [61, 62]. In conclusion, lipid disorders provide a large "lipid pool" for the development of HCC, and specific mechanisms of aberrant lipid metabolism in hepatic regulation could greatly improve the efficiency of immunotherapy, but clinicians are not yet able to apply molecular profiles to guide dosing, so LD-related factors were summarized. Therefore, by summarizing LD-related factors, it is imperative to understand the factors that are critical for OS.

In this study, 124 LD-associated genes were searched based on the available medical literature and subsequently differentially expressed in three genes (ANXA2, CKAP4, IRAK1) in the TCGA and ICGC datasets, and survival analysis and time-dependent ROC analysis showed the same survival trends and ability to predict survival probabilities in both datasets, but prognostic models for 1-,3-, and 5-year survival was not a valid guide. Further functional analysis in the TCGA dataset suggested ER stress is an important component and function of LD, which was also validated in the CRISPR/cas9 screening library. Therefore, a 10-genes risk signature associated with LD and ER stress was created from the Genecards website based on survival and COX regression analyses and showed that this risk model is an independently identified factor for prognosis that is associated with regulation of the tumor immune microenvironment, specifically M2-type macrophage infiltration and TIM-3 expression.

First, genes associated with LD in the literature search, and three genes were screened by differential gene expression and survival analysis with TCGA and ICGC datasets. These genes were expressed at high levels in tumor tissues, suggesting poor survival, but further prognostic risk modeling showed that these genes were not superior in predicting survival probability. This may be explained by two reasons: 1. the different regions and prevalence of HCC patients in the two databases, as well as the different variations in life and dietary habits, prevent homogenization of the analysis. 2. the current molecular mechanisms of LD are mainly focused on metabolic diseases [63], while malignancies have been studied relatively singularly [64], especially the specificity of lipid metabolism in HCC [65], leading to gene inconsistent expression and survival analyses.

Considering that this risk model from literature data has a strong predictive power for survival in HCC patients and its limitations, the functional mechanisms of these genetic features were further evaluated. Functional analysis revealed important effects on ER stress response and ubiquitin-dependent ER-associated degradation (ERAD) actions. These findings suggest that LD-associated genes are closely related to the process of ER stress activation. ER stress is triggered when there is a mismatch between the ability of the ER to process proteins and its ability to fold proteins [66]. Acute ER stress primarily causes cellular damage, whereas chronically ER stress can be a hallmark of many diseases, even tumors [67]. It is known that ER stress state induced signaling and regulation can enhance tumor proliferation, angiogenesis, vaccine escapes and tolerance to radiotherapy [68]. In HCC, ER stress leads to activation of corresponding genes due to rapid proliferation of cancer cells resulting in ischemic hypoxia and oxidative stress in tumor tissue. Therefore, aberrant ER stress gene expression may be of prognostic value in HCC cases.

Furthermore, Hep3B and SNU398 cell screens were constructed by CRISPR/cas9 knockout library, and functional enrichment analysis by GO and KEGG revealed that these genes were extremely strongly associated with ER stress, similar to the results of functional enrichment methods obtained from literature searches. CRISPR/cas9 technology has significant promise for identifying important genes when screening genomic functions in biologic processing in varied biologic models. It is already being used in a genome-wide context as an alternate screen of RNA interferences to target alterations in gene function [69]. The promise of CRISPR screens for function in genomics is to help researchers discover one or more gene functions and potentially enable their alteration, for example to conduct cancer research such as molecular mechanistic analyses and therapy explorations. CRISPR technology has been used in viral infections [70], genetic diseases [71], antibiotic resistant bacteria [72].

Risk score is often a common method for developing meaningful signatures. The main purpose of analyzing genes screened from the TCGA dataset is to understand whether LD and ER stress gene risk scores are of value. ER stress-related risk scores have been less studied, and two studies of glioma simultaneously illustrate that activation of ER stress genes has important implications on patient prognosis and immune status [73, 74]. Therefore, the results screened by comparing the two datasets may be able to provide some value to clinicians for decision making.

Models using risk score can accurately predict not only the outcome, but also distinguish between different clinicopathological features. Time-dependent ROC analysis showed that risk characteristics performed well in predicting 1-, 3-, and 5-year survival of HCC patients in the TCGA dataset. Survival analysis confirmed that risk scores accurately predicted patient survival, and similar results were found in the ICGC dataset.

In the TCGA dataset, the results of clinicopathologic features are suggestive of HBV infection, deeper local infiltration, higher histologic grade, and higher risk scores in patients with advanced HCC. These suggestive risk features are usually associated with poor prognosis and allow accurate differentiation of varying clinicopathological features in HCC patients. Currently, despite the new direct-acting antiviral drug treatments now available, epigenetic dysregulation of residual risk after HCV infection or after cure induces alterations in the host cell transcriptome [75] and direct carcinogenesis of viral infection [76], as well as on the accumulation of hepatocytes in the inflammatory process and the generation of inflammatory oxygen species [76], leading to a process that displays dysregulation via HCV infection, called cancer marker [76]. However, there was no difference in risk scores between HCV infection and those without viral infection, contrary to previous studies [77]. A possible explanation is that HCV-infected patients have a relatively small sample size and do not show a trend of poor prognosis. In addition, stage IV patients had the lowest risk scores because only five stage IV patients were available in the TCGA dataset, which would not better explain the full characteristics.

Immunological and inflammatory reactions together with biological processes of biological synthesis and biodegradation were enclosed in the higher risk group. High expression of TIM-3 in tumor immune checkpoints and M2-type macrophages was more pronounced in the high-risk group compared to the low-risk score. TIM-3 plays an immunosuppressive role in the immune microenvironment, not only inducing depletion of CD8 + T cells [78], but also promoting activation of Treg cell [79] and massive proliferation of MDSCs [80], implying that LDs upregulation may inhibit the antitumor immune response in HCC. Furthermore, consider that immune cell infiltrates are an essential element of the immunological microenvironment [81], further studies investigated the relationship between risk scores and immunocells, like macrophages, dendritic cells, B cells, CD4 + and CD8 + T cells, and neutrophils. This suggests that lipid aggregation and activation of ER stress can regulate changes in the immune microenvironment by affecting patient prognosis.

To exploit the full potential of the risks model, a nomogram was prepared to combine risk characteristics, age, sex, race, histological class, T and TNM stage. The calibration curves based on the TCGA dataset demonstrated good predictive performance. Thus, the established LD-associated and ER stress gene risk signature can predict 1-, 3-, and 5- OS probabilities for individualized treatment strategies.

The comparison and novelty of this paper with the current studies is reflected in three aspects. Firstly, previous studies [82] showed the analysis of prognostic characteristics of lipid-related genes in the TCGA database for pancreatic cancer in literature searches and found that some genetic mutations were prompt for worse prognosis. In this paper, we used a similar method and conducted the prognostic analysis of lipid-related genes using the TCGA database and the ICGC database as a training cohort and a validation cohort, respectively. Unfortunately, negative results were obtained in this paper. This shows the need for validation of the results obtained by database analysis. Secondly few studies [73, 74] have shown that activation of ER stress genes has an important impact on the prognosis and immune status of cancer patients, and this paper combines both ER stress and LDs through functional analysis of public databases, which is less common in previous studies. This combined approach accurately refined the study direction and optimized the study population. Finally, our institute [38] constructed an HCC knockout library by CRISPR/cas9 technology, and this paper verified the strong association between LDs and ER stress, which was rare in previous CRISPR/cas9-related publications [69, 70, 72] and increased the scope of CRISPR/cas9 utilization.

The important strengths include the association of LD and ER stress, and for the first time in HCC, common genes associated with LD and ER stress have been analyzed and a 10-gene risk model has been developed that can effectively predict prognostic and biological characteristics. Moreover, this paper validated ER stress as the strongest correlate of LDs by CRISPR/cas9 technology in HCC cell lines. In addition, it was found that the currently available literature supported LD-related genes do not establish consistent prognostic features in TCGA and ICGC databases. However, our study has a number of limitations. This risk model was not screened at this step due to too few genes were obtained by multifactorial COX regression analysis. Although the results were similar in distinguishing survival differences, clinicopathological features and immunological events in both datasets, the accuracy of such risk model in other datasets remains to be investigated.

## Conclusion

In the present study, the prognosis of HCC patients was assessed by risk scores for LD-related and ER stress genes constructed by functional analysis to determine the significance of ER stress on lipid function, despite the failure of genetic risk models obtained from the existing literature. This risk model suggested that high risk scores were related to worse prognosis, closely related to M2 macrophage and TIM-3 expression. Thus, these provide a certain molecular basis for the prognostic prediction and biological characterization of HCC patients, and add a valuable reference direction for molecular studies. Especially in patients with lipid-associated HCC, this risk score can be a more effective and visual indication of the prognostic impact. we can design appropriate targeted drugs.

## Supplementary Information

**Additional file 1: Fig. S1.** Flow chart of literature search. **Fig. S2.** Gene heat map in HCC patients. **Fig. S3.** Gene expression differences in the TCGA dataset. **Fig. S4.** Differential expression of proteins in the TCGA dataset. **Fig. S5.** Prognosis value for overall survival in the TCGA dataset. **Fig. S6.** Gene expression differences in the ICGC dataset. **Fig. S7.** Prognosis value for overall survival in the ICGC dataset. **Fig. S8.** Univariate COX regression analysis. **Fig. S9.** The expression pattern of the 3 genes. **Fig. S10.** The prognostic value of the ER stress-related signature in TCGA and CGGA datasets. **Fig. S11.** The prognostic value of the ER stress-related signature in TCGA dataset. **Fig. S12.** Correlation between the 10 genes and their genetic alteration status. **Fig. S13.** The expression profiles of the proteins. **Fig. S14.** Association between the signature and clinicopathologic features in ICGC datasets. **Fig. S15.** Relationship betweenthe risk signature and immune checkpoints. **Fig. S16.** Forest plot of the univariate and multivariate Cox regressionanalysis in the ICGC cohorts. **TableS1.** 124 factors were identified as lipiddroplet-associated factors by literature search.

## Data Availability

Genecards (https://www.genecards.org/); TCGA (https://portal.gdc.com); ICGC (https://dcc.icgc.org/releases/current/Projects); UALCAN (http://ualcan.path.uab.edu/); *ACLBi* (http://www.acbi.com); DAVID (http://david.ncifcrf.gov/); cBioPortal for Cancer Genomics (https://www.cbioportal.org/); HPA website (https://www.proteinatlas.org/).

## Abbreviations

ER: Endoplasmic reticulum
HCC: Hepatocellular carcinoma
LD: Lipid droplet
TCGA: The Cancer Genome Atlas
ICGC: International Cancer Genome Collaboratory
LASSO: Last absolute shrinkage and selection operator
TIM-3: T cell immunoglobulin and mucin-3
HBV: Hepatitis B virus
NAFLD: Non-alcoholic Fatty Liver Disease
NASH: Non-alcoholic Steatohepatitis
DEGs: Differentially expressed genes
CPTAC: Clinical Proteomic Tumor Analysis Consortium
OS: Overall survival
DAVID: The Database for Annotation, visualization and integrated discovery
GO: Gene ontology
KEGG: Kyoto encyclopedia of genes and genomes
AIC: Akaike information criterion
HPA: The human protein atlas
MDSCs: Myeloid-derived suppressor cells
T: Treg regulatory
NK: Natural killer
TMB: Tumor mutation burden
MSI: Microsatellite instability
SD: Standard deviation
ANXA2: Annexin A2
BCAP31: B cell receptor-associated protein 31
CKAP4: Cytoskeleton-associated protein 4
HSD17B13: Hydroxysteroid 13
IRAK1: Interleukin-1 receptor-associated kinase
SQLE: Squalene epoxidase, STC2 stanniocalcin 2
SPP1: Secreted phosphoprotein 1
PSMD1: Proteasome 26S subunit, non-ATPase 1
PRDX1: Peroxiredoxin 1
LPCAT1: Lysophosphatidylcholine acyltransferase 1
KPNA2: Karyopherin subunit alpha 2
HDGF: Heparin binding growth factor
G6PD: Glucose-6-phosphate dehydrogenase
DYNC1LI1: Dynein cytoplasmic 1 light intermediate chain 1
B3GAT3: Beta-1,3-glucuronyltransferase 3
IGF: Insulin-like growth factor
NLSs: Nuclear localization sequence
PD-1: Programmed death 1
CTLA-4: Cytotoxic T lymphocyte-associated antigen 4
LAG-3: Lymphocyte-activation gene 3
IRE1α: Inositol-requiring enzyme 1 alpha
PERK: Protein kinase RNA like endoplasmic reticulum kinase
ERAD: ER-associated degradation
