# Supplementary material for: Characterization of a lipid droplet and endoplasmic reticulum stress related gene risk signature to evaluate the clinical and biological value in hepatocellular carcinoma
Source: Lipids Health Dis. 2022 Dec 29;21:146. doi: 10.1186/s12944-022-01759-y (PMC9798721; doi:10.1186/s12944-022-01759-y)
Supplement: Supplementary file 1 — Additional file 1: Fig. S1. Flow chart of literature search. Fig. S2. Gene heat map in HCC patients. Fig. S3. Gene expression differences in the TCGA dataset. Fig. S4. Differential expression of proteins in the TCGA dataset. Fig. S5. Prognosis value for overall survival in the TCGA dataset. Fig. S6. Gene expression differences in the ICGC dataset. Fig. S7. Prognosis value for overall survival in the ICGC dataset. Fig. S8. Univariate COX regression analysis. Fig. S9. The expression pattern of the 3 genes. Fig. S10. The prognostic value of the ER stress-related signature in TCGA and CGGA datasets. Fig. S11. The prognostic value of the ER stress-related signature in TCGA dataset. Fig. S12. Correlation between the 10 genes and their genetic alteration status. Fig. S13. The expression profiles of the proteins. Fig. S14. Association between the signature and clinicopathologic features in ICGC datasets. Fig. S15. Relationship betweenthe risk signature and immune checkpoints. Fig. S16. Forest plot of the univariate and multivariate Cox regressionanalysis in the ICGC cohorts. TableS1. 124 factors were identified as lipiddroplet-associated factors by literature search. [file 12944_2022_1759_MOESM1_ESM.docx]

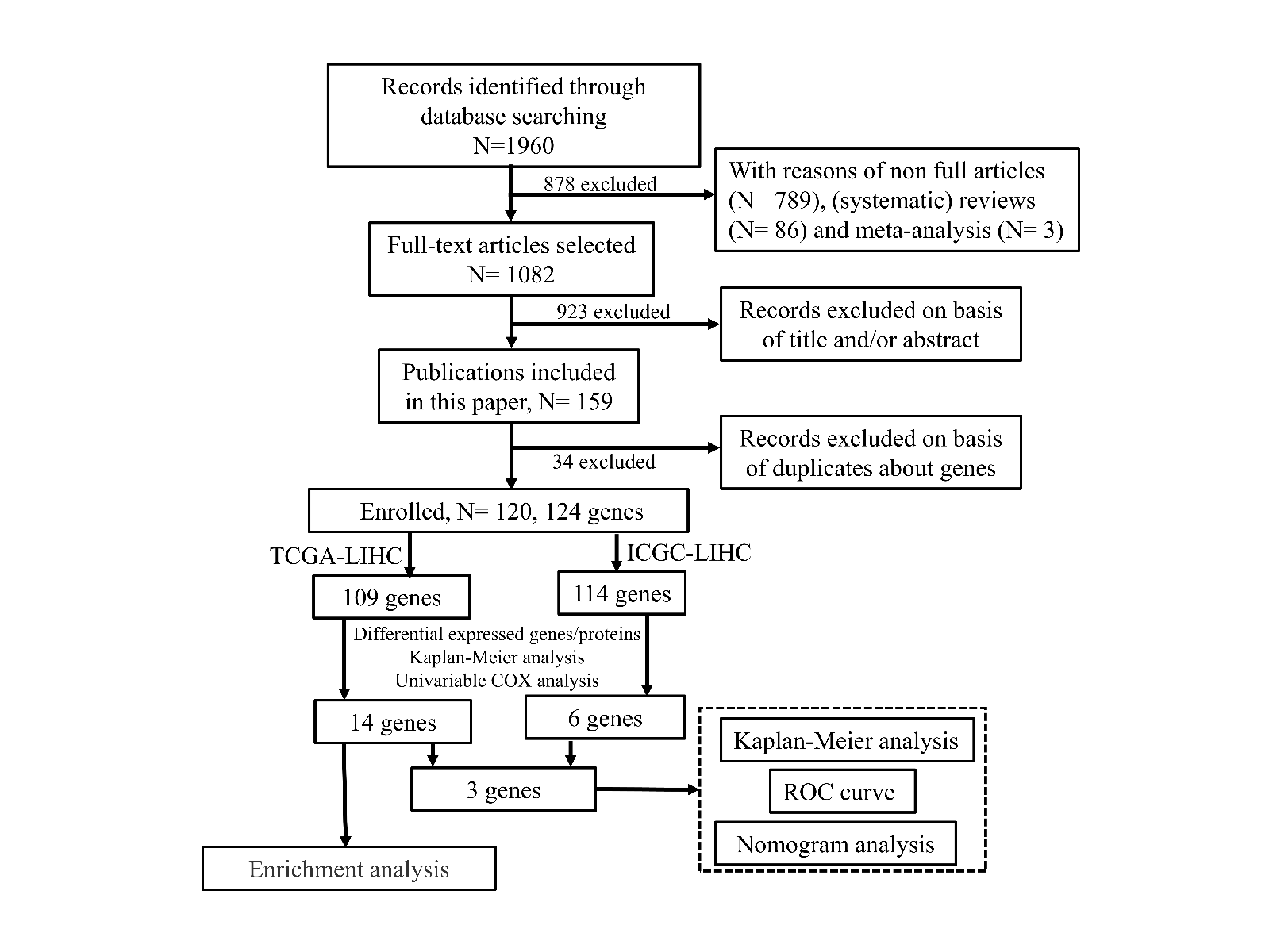


Fig. S1 Flow chart of literature search.


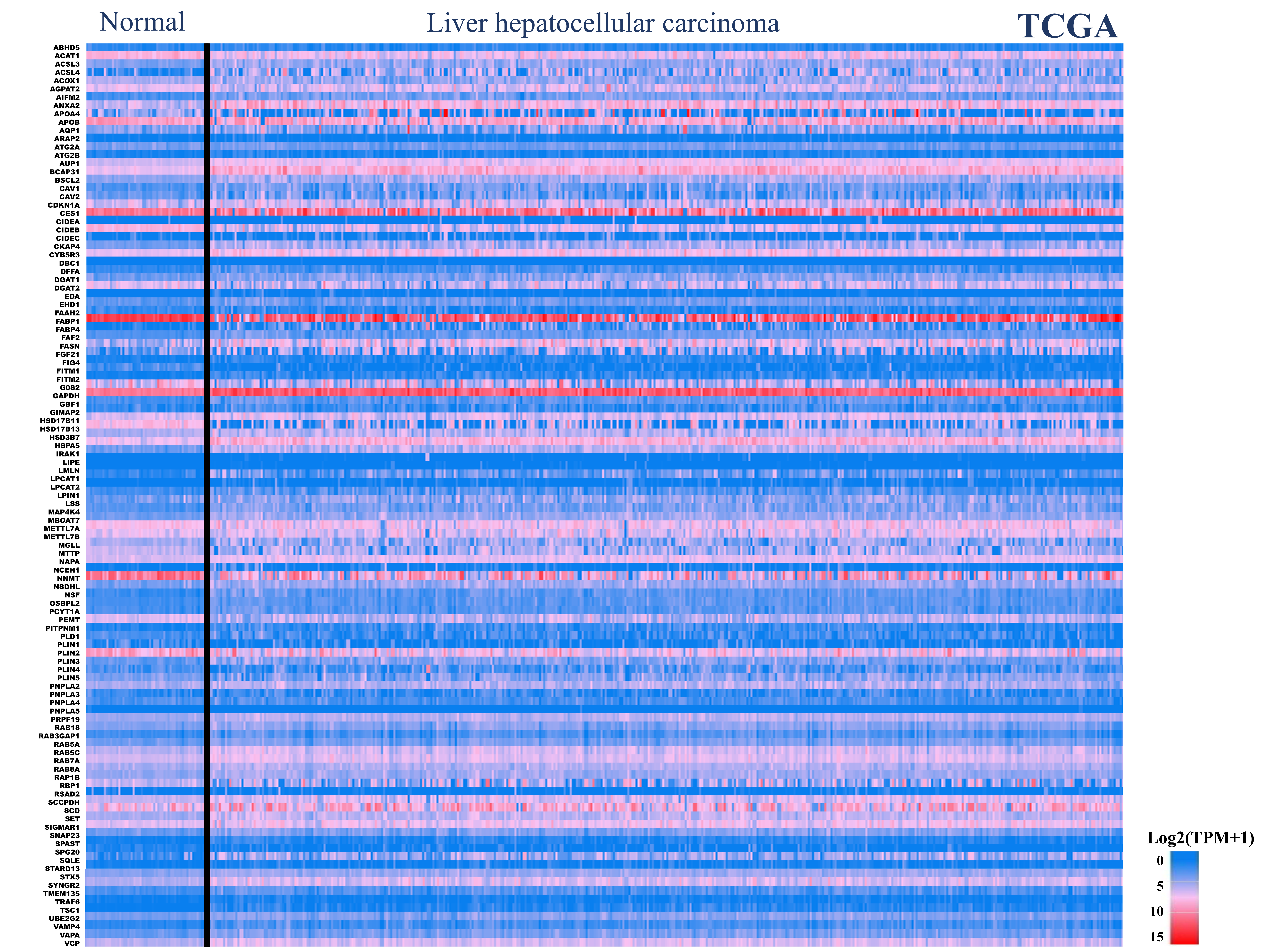


Fig. S2 Gene heat map from literature search of 109 genes in HCC patients based on TCGA database.


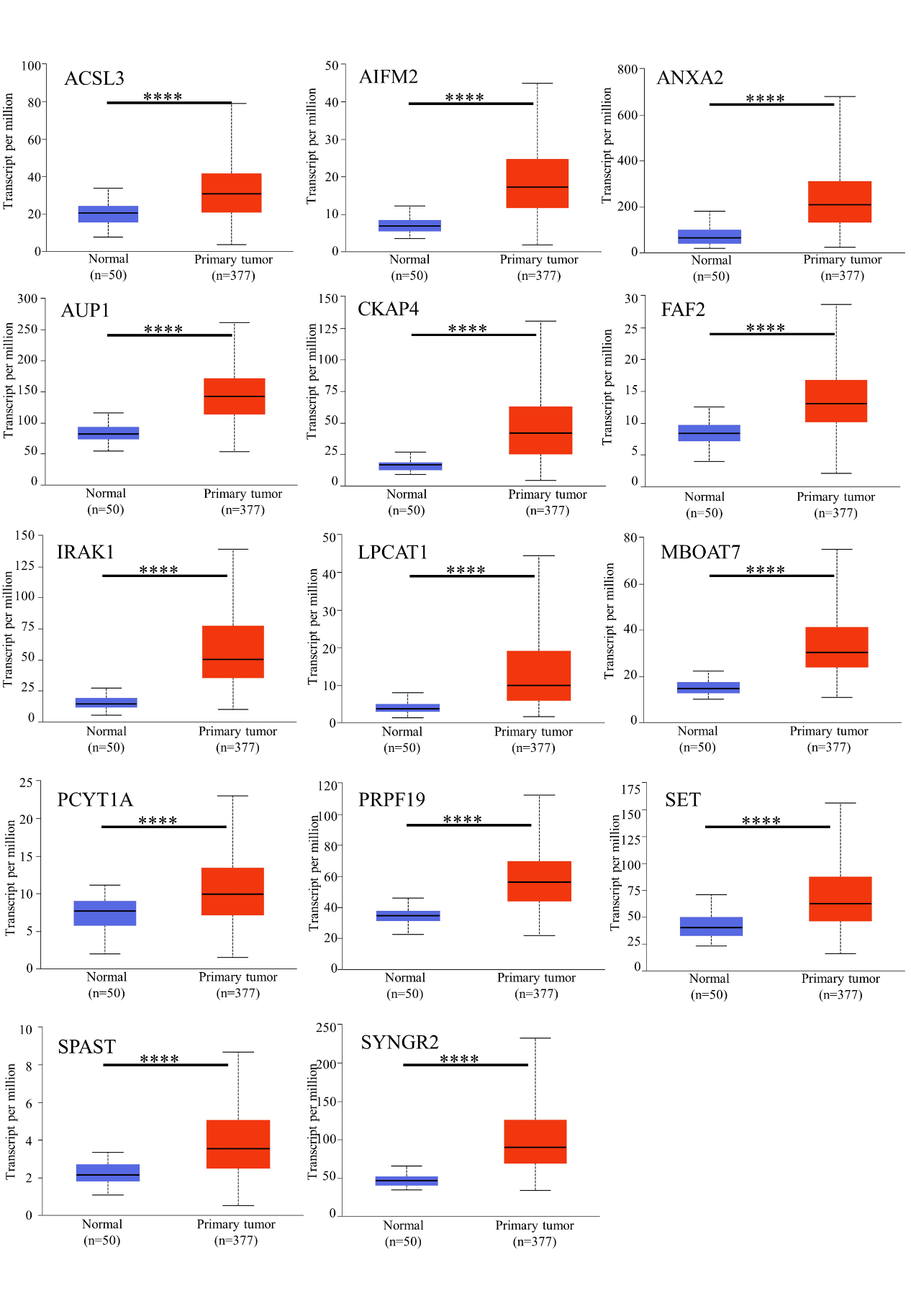


Fig. S3 Gene expression differences of 14 genes in the TCGA dataset. **** *P*＜0.001, *** *P*＜0.01, ** *P*＜0.05, * *P*≥0.05


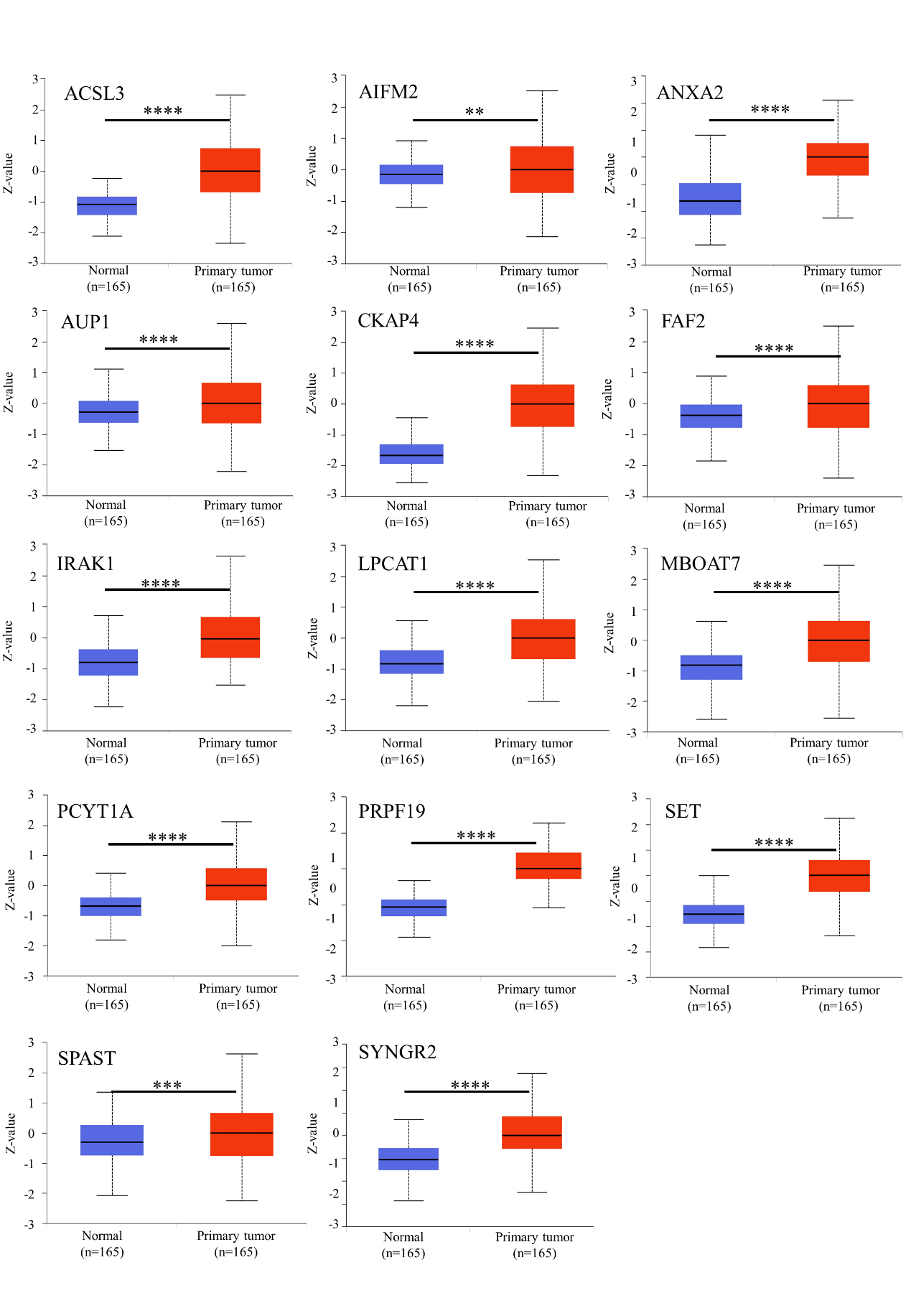


Fig. S4 Differential expression of proteins encoded by 14 genes in the TCGA dataset. **** *P*＜0.001, *** *P*＜0.01, ** *P*＜0.05, * *P*≥0.05


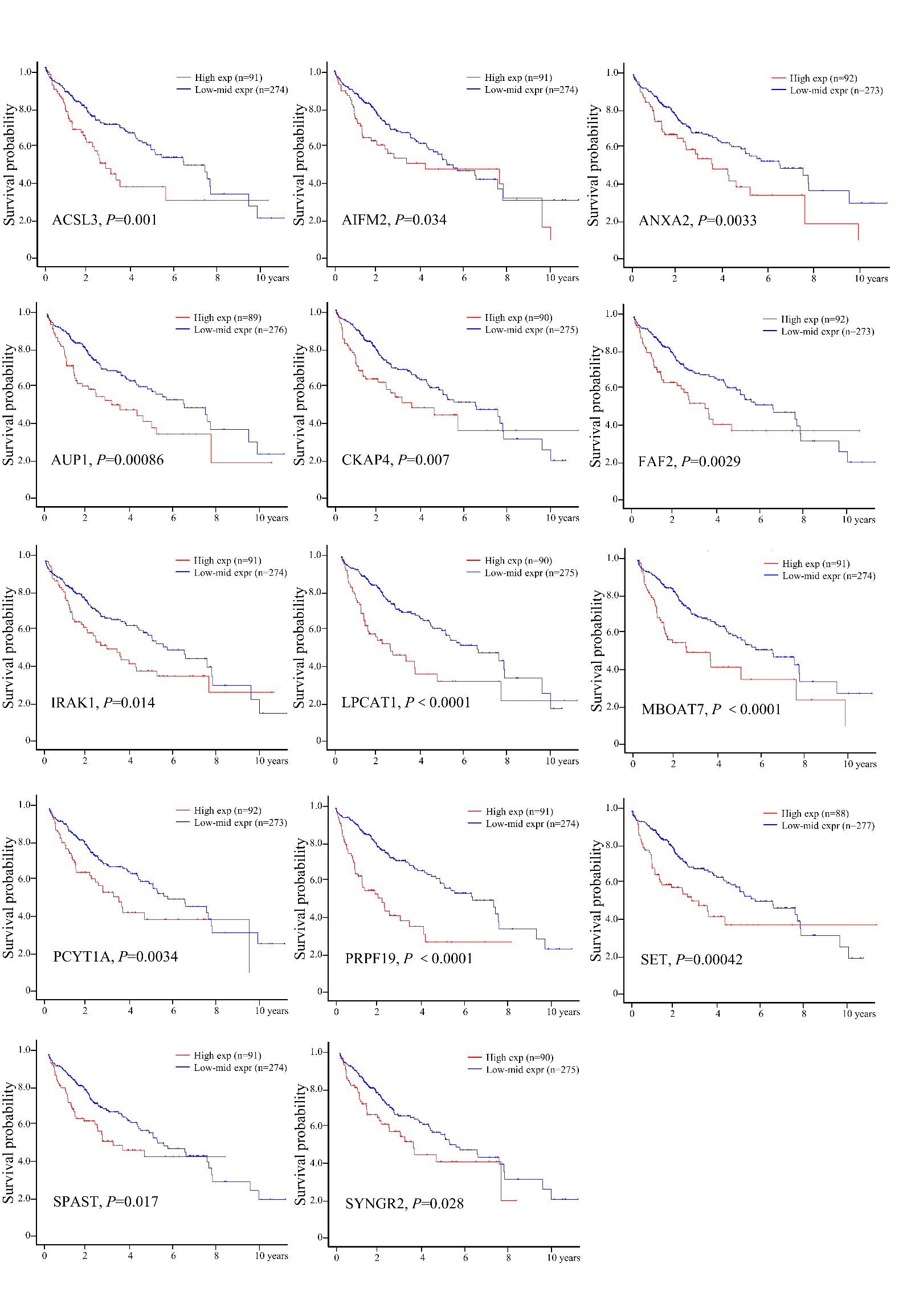


Fig. S5 Prognosis value for overall survival in 14 genes in the TCGA dataset.


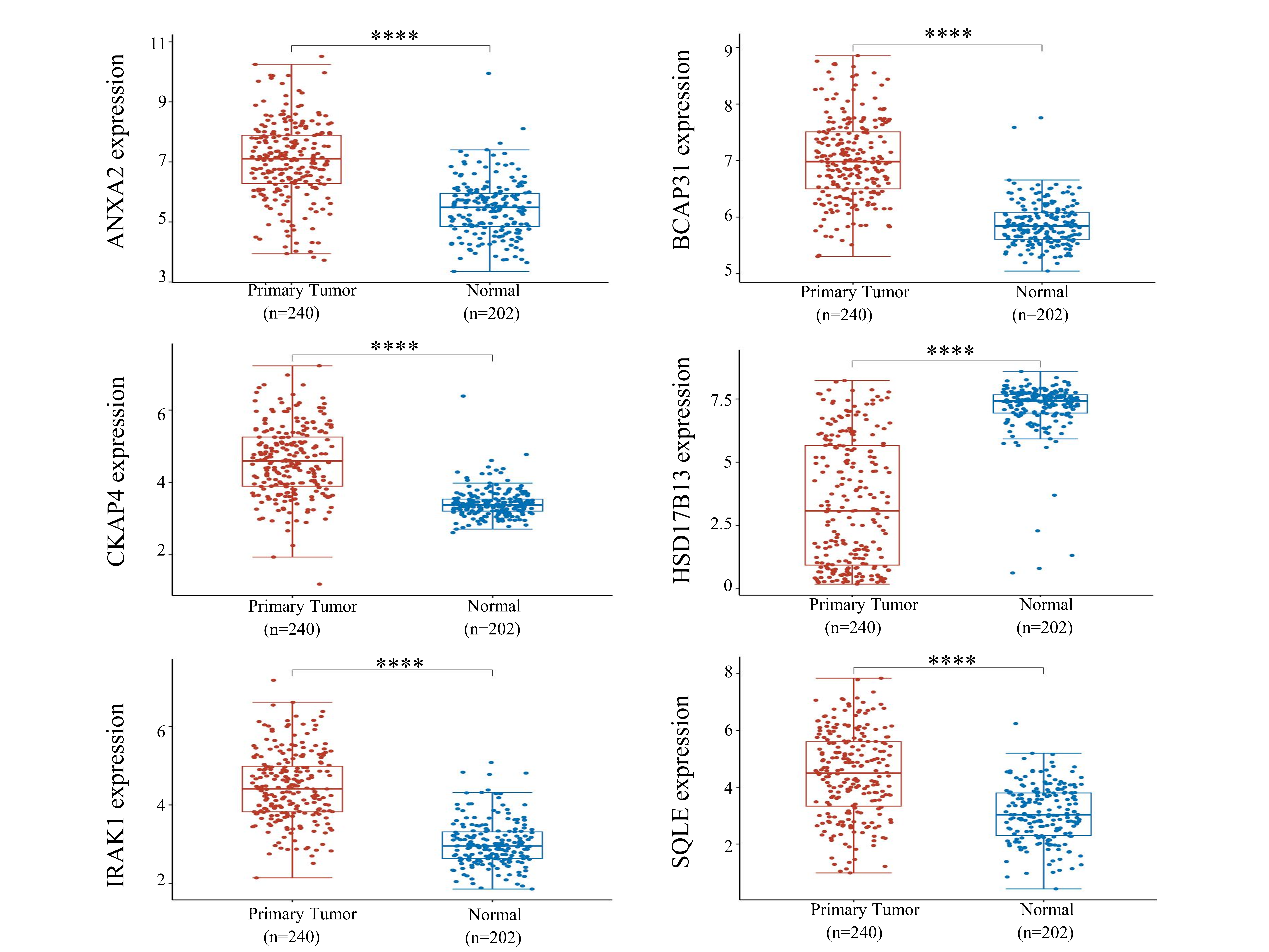


Fig. S6 Gene expression differences of 6 genes in the ICGC dataset. **** *P*＜0.001, *** *P*＜0.01, ** *P*＜0.05, * *P*≥0.05


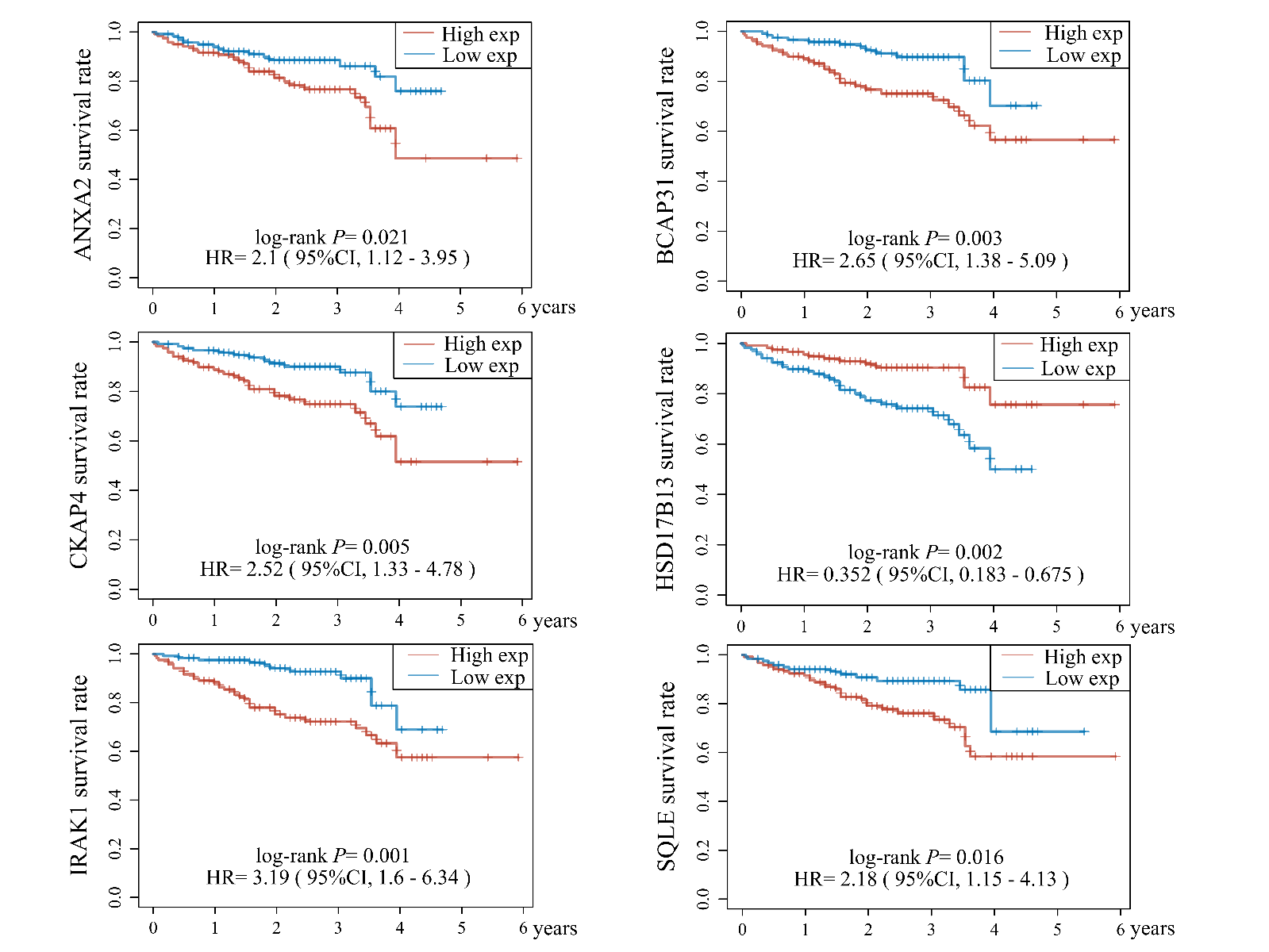


Fig. S7 Prognosis value for overall survival in 6 genes in the ICGC dataset.


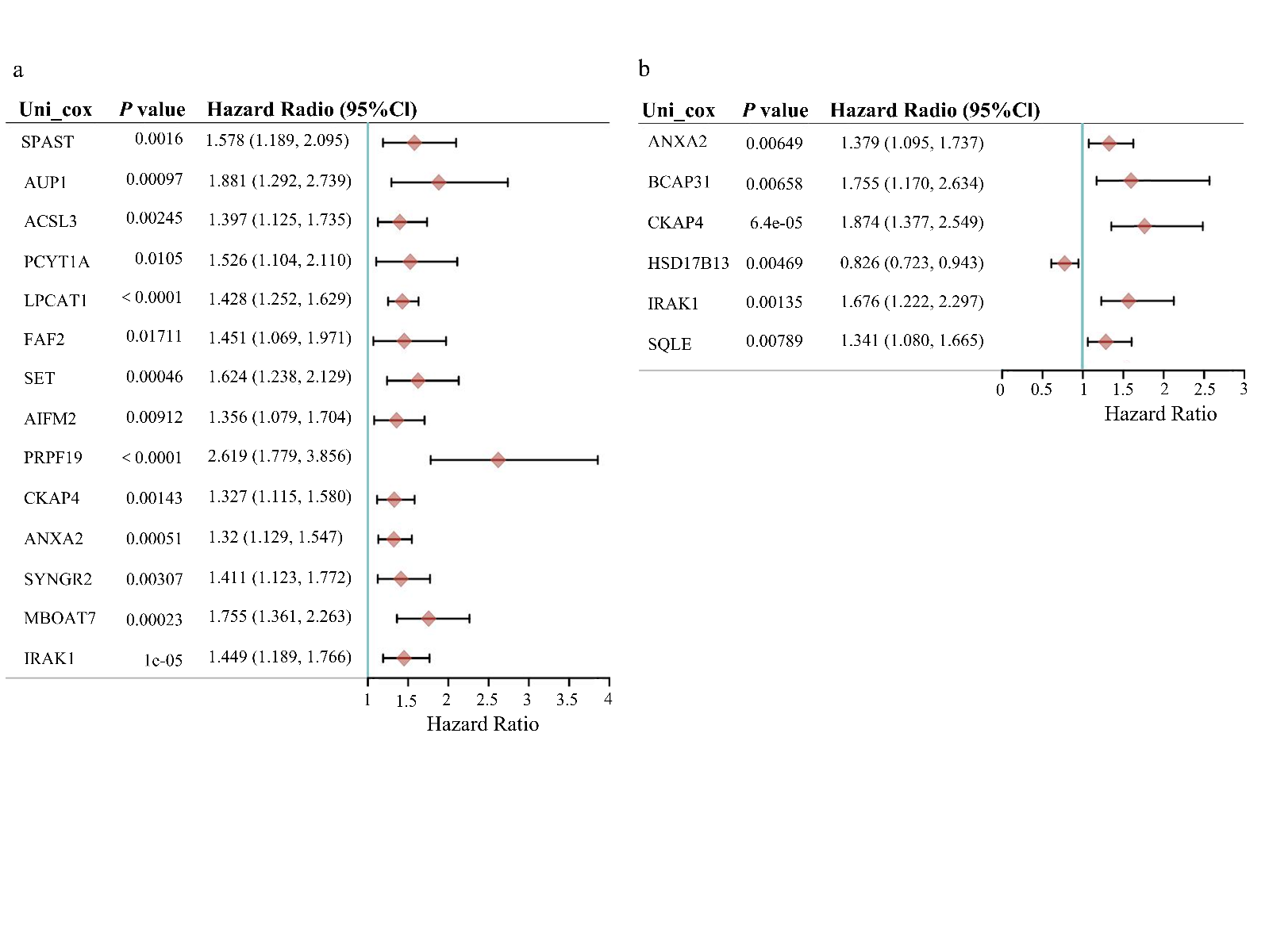


Fig. S8 Univariate COX regression analysis of 14 genes in the TCGA dataset (a) and 6 genes in the ICGC dataset (b).


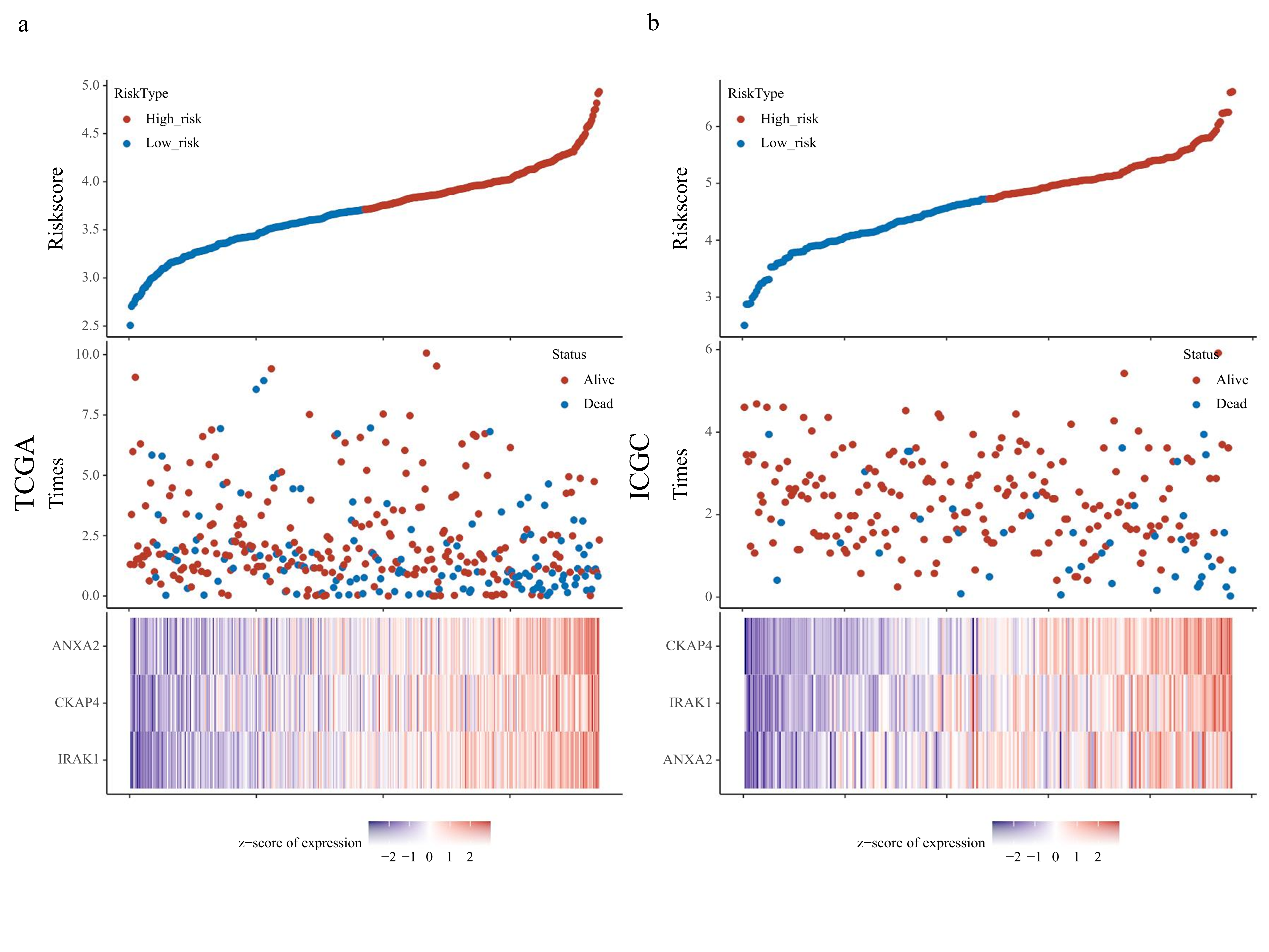


Fig. S9 The expression pattern of the 3 (ANXA2, CKAP4, IRAK1) genes. (a) Risk score of the 3-gene signature in the TCGA cohort. (b) Risk score of the 3-gene signature in the ICGC cohort.


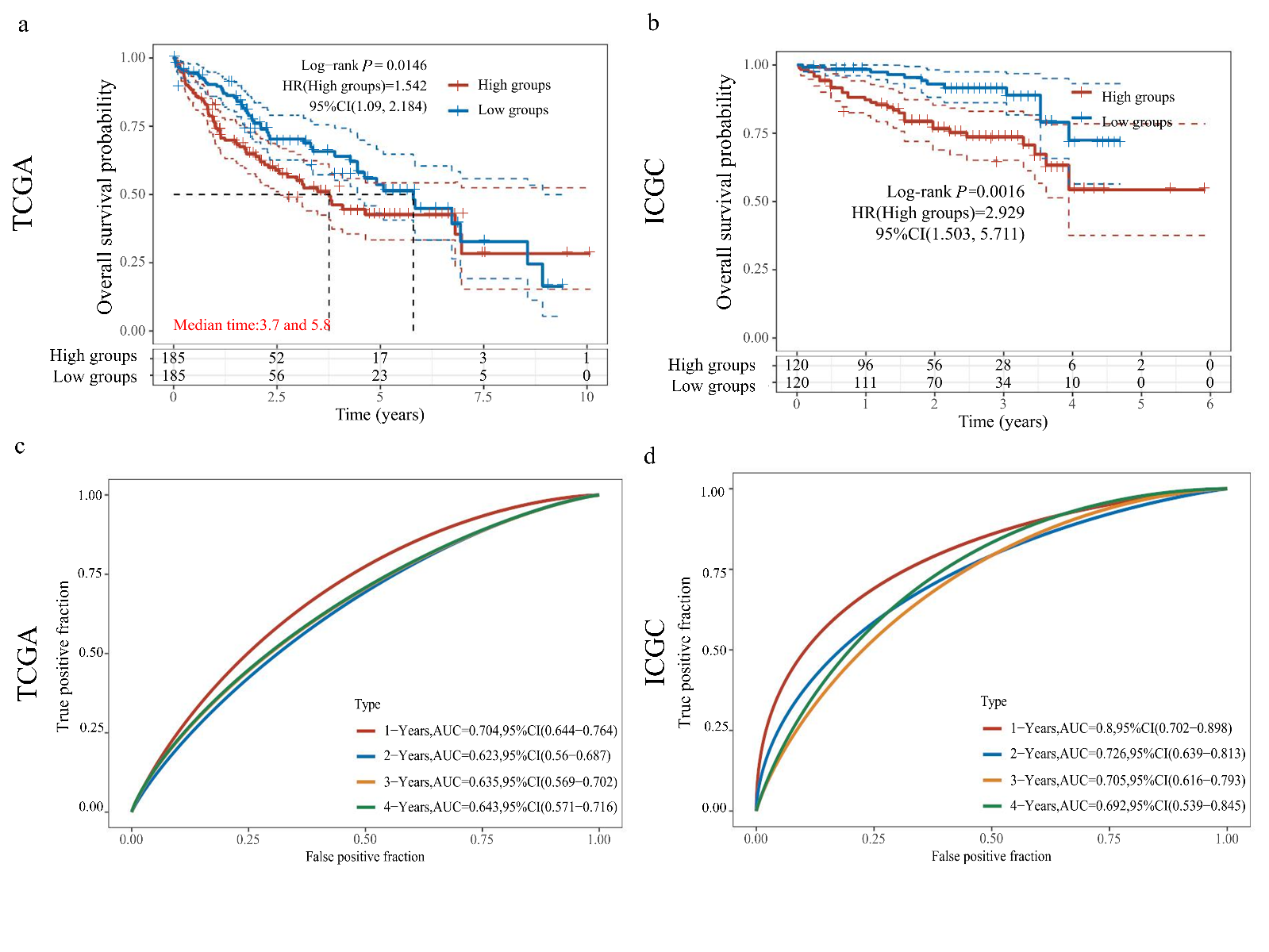


Fig. S10 The prognostic value of the ER stress-related signature in TCGA and CGGA datasets. (a, b) K-M survival analyses of the risk signature in HCC patients. (c, d) The time ROC curve analyses were performed to predict 1-, 2-, 3-, and 4-year OS according to risk score in the TCGA and ICGC datasets.


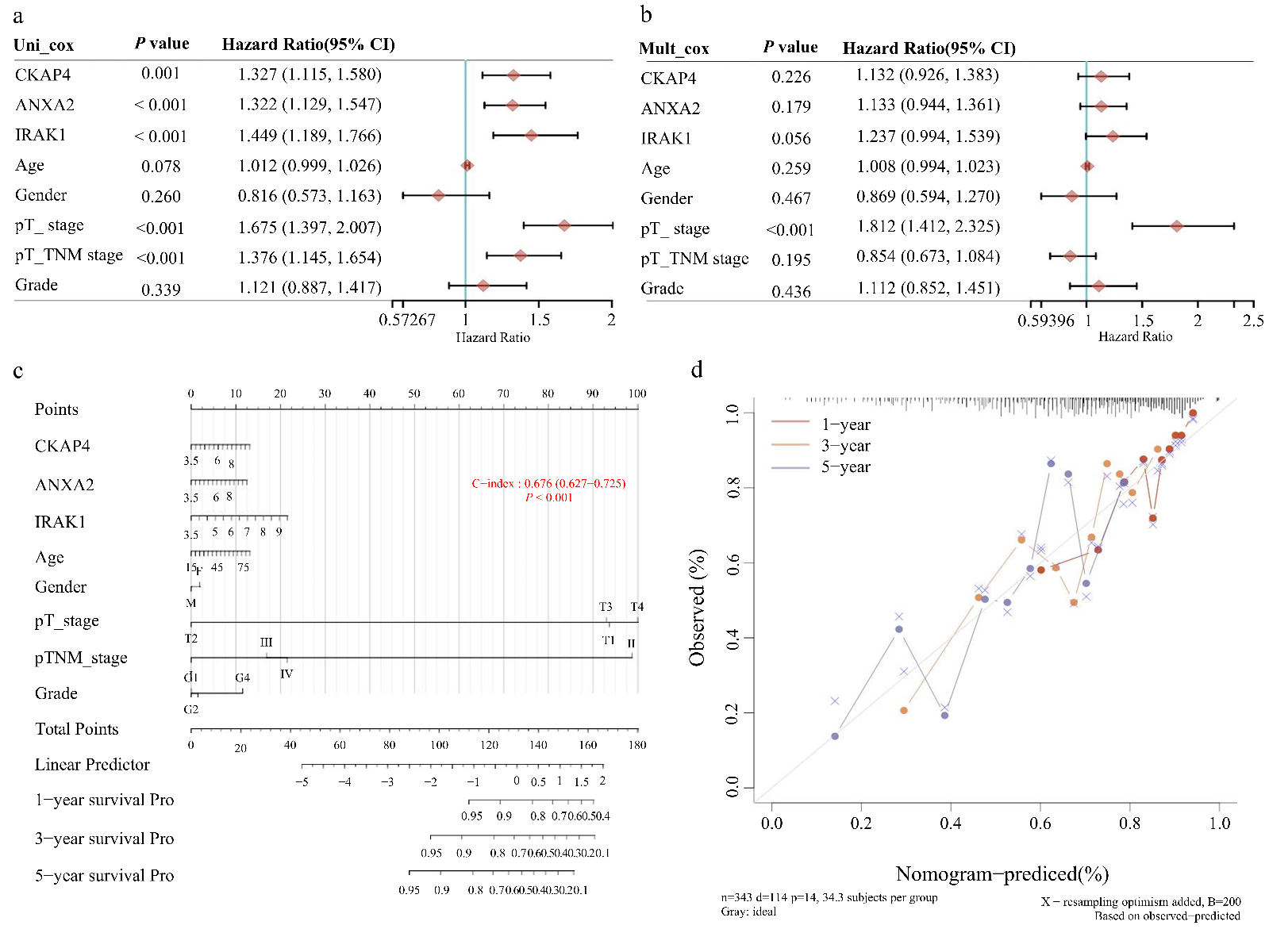


Fig. S11 The prognostic value of the ER stress-related signature in TCGA dataset. (a, b) Univariate (a) and multivariable (b) analyses of 3 genes in the TCGA dataset. (c, d) The nomogram (c) and the calibration curve (d) analyses were performed to predict 1-, 3-, and 5-year OS according to risk score.


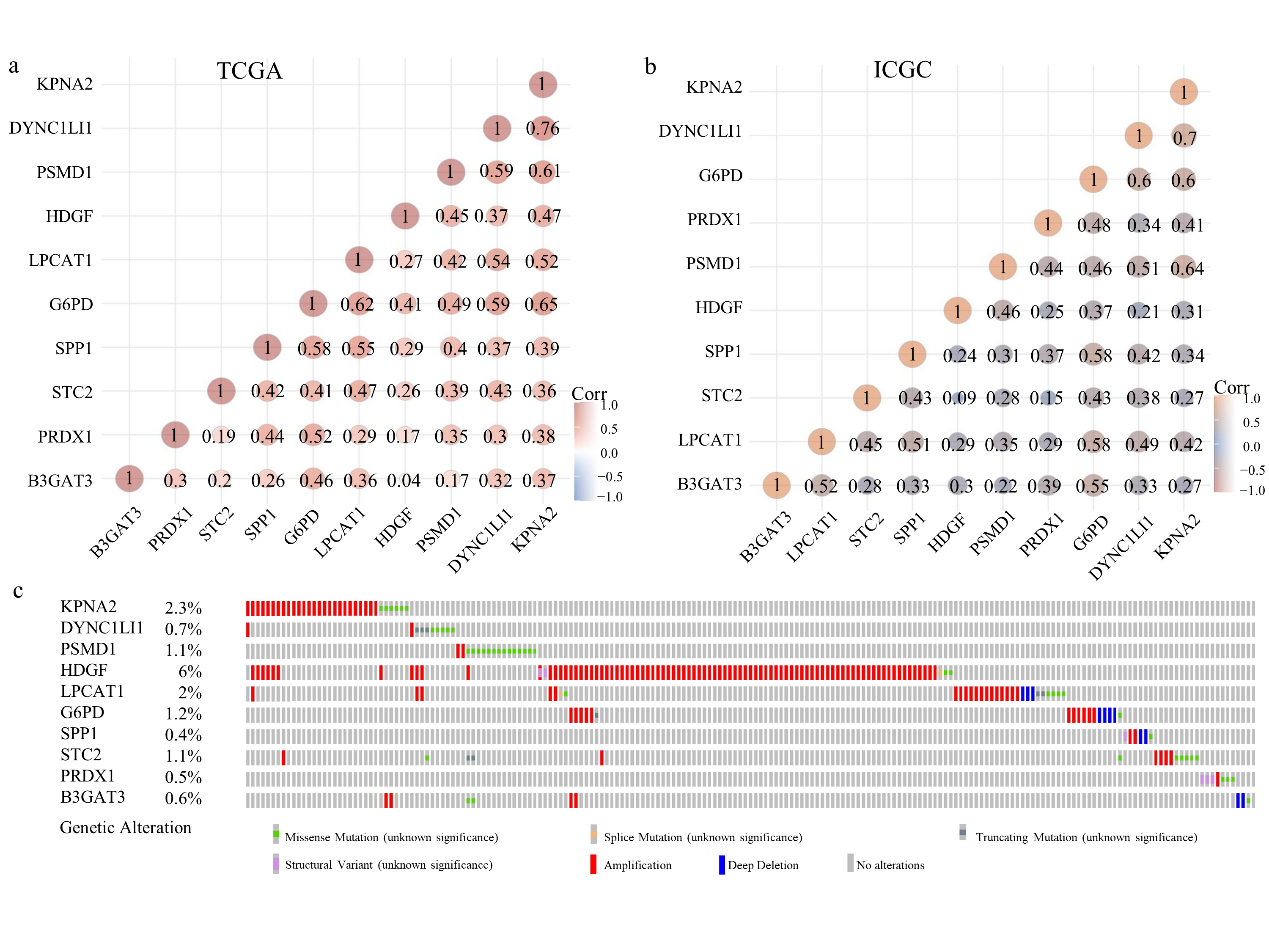


Fig. S12 Correlation between the 10 genes and their genetic alteration status. (a-b) Spearman correlation analysis of 10 ER stress-related genes in the TCGA and ICGC datasets. (c) Genetic alteration of the 10 genes in the TCGA HCC cohort.


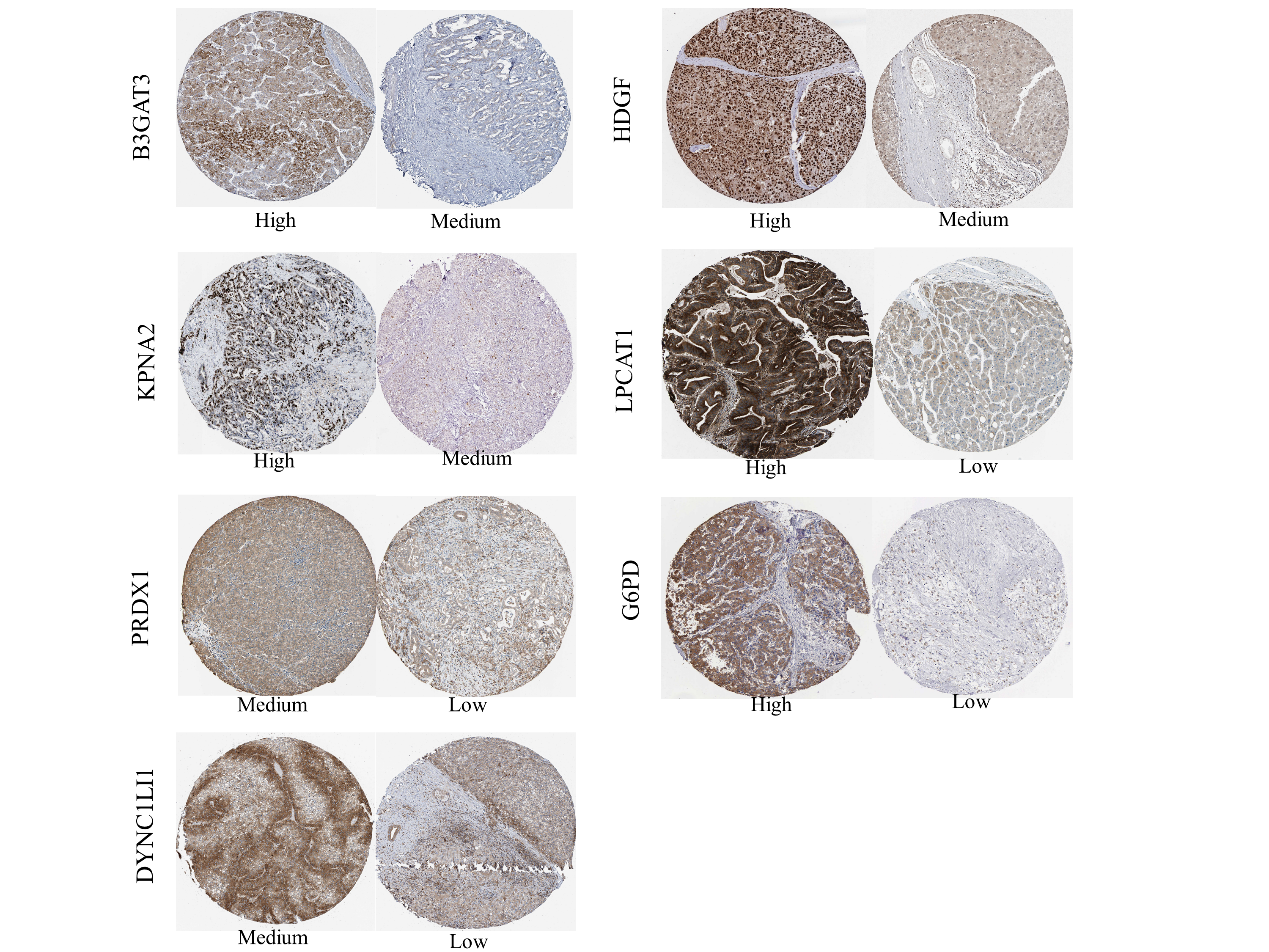


Fig. S13 The expression profiles of the proteins encoded by B3GAT3, KPNA2, PRDX1, DYNC1LI1, HDGF, LPCAT1 and G6PD in HCC tissues using clinical specimens from the Human Protein Profiles.


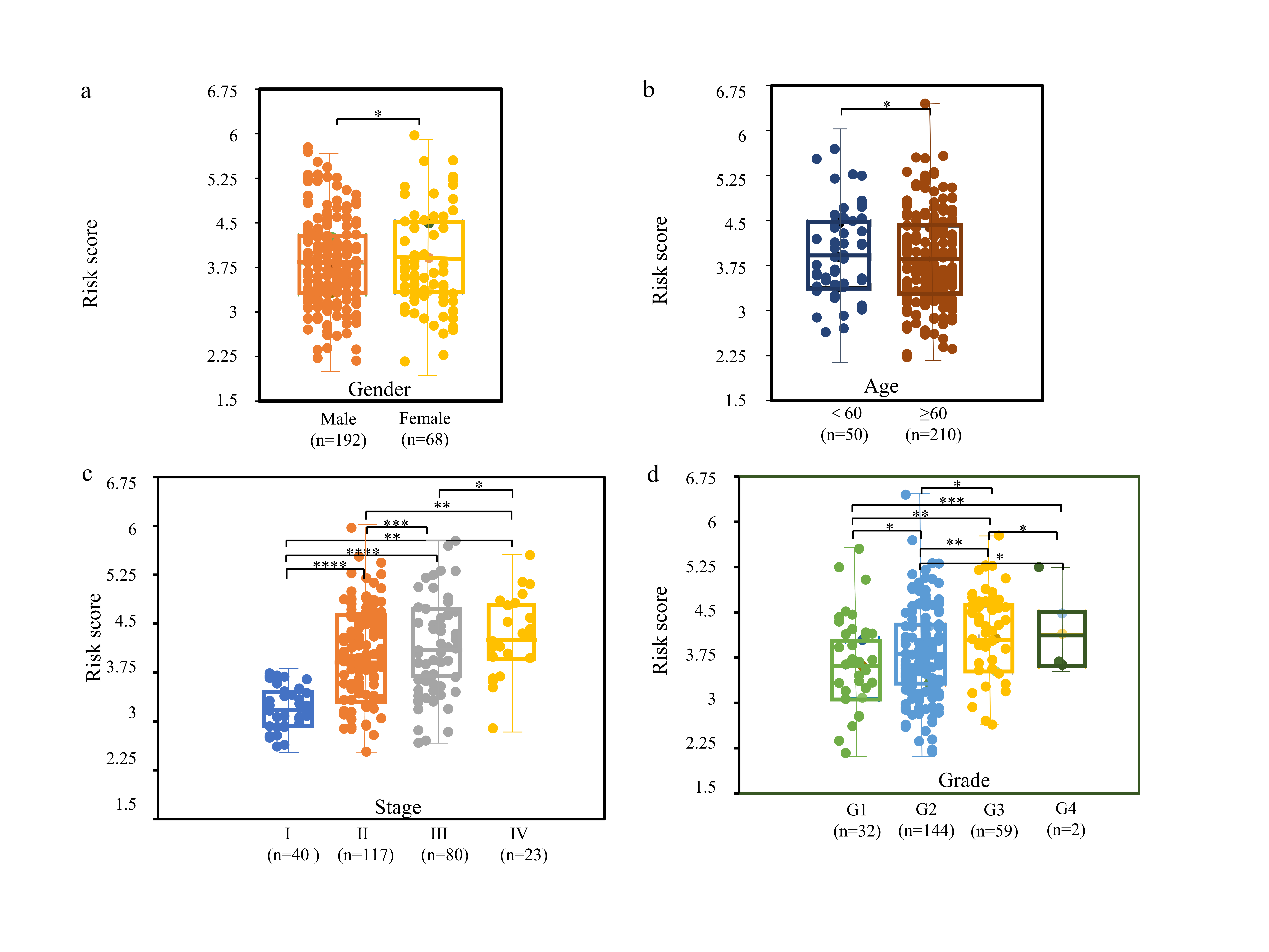


Fig. S14 Association between the signature and clinicopathologic features in ICGC datasets. The association between risk score and gender (a), age (b), TNM stage (c) and grade (d) of HCC patients. **** *P*＜0.001, *** *P*＜0.01, ** *P*＜0.05, * *P*≥0.05


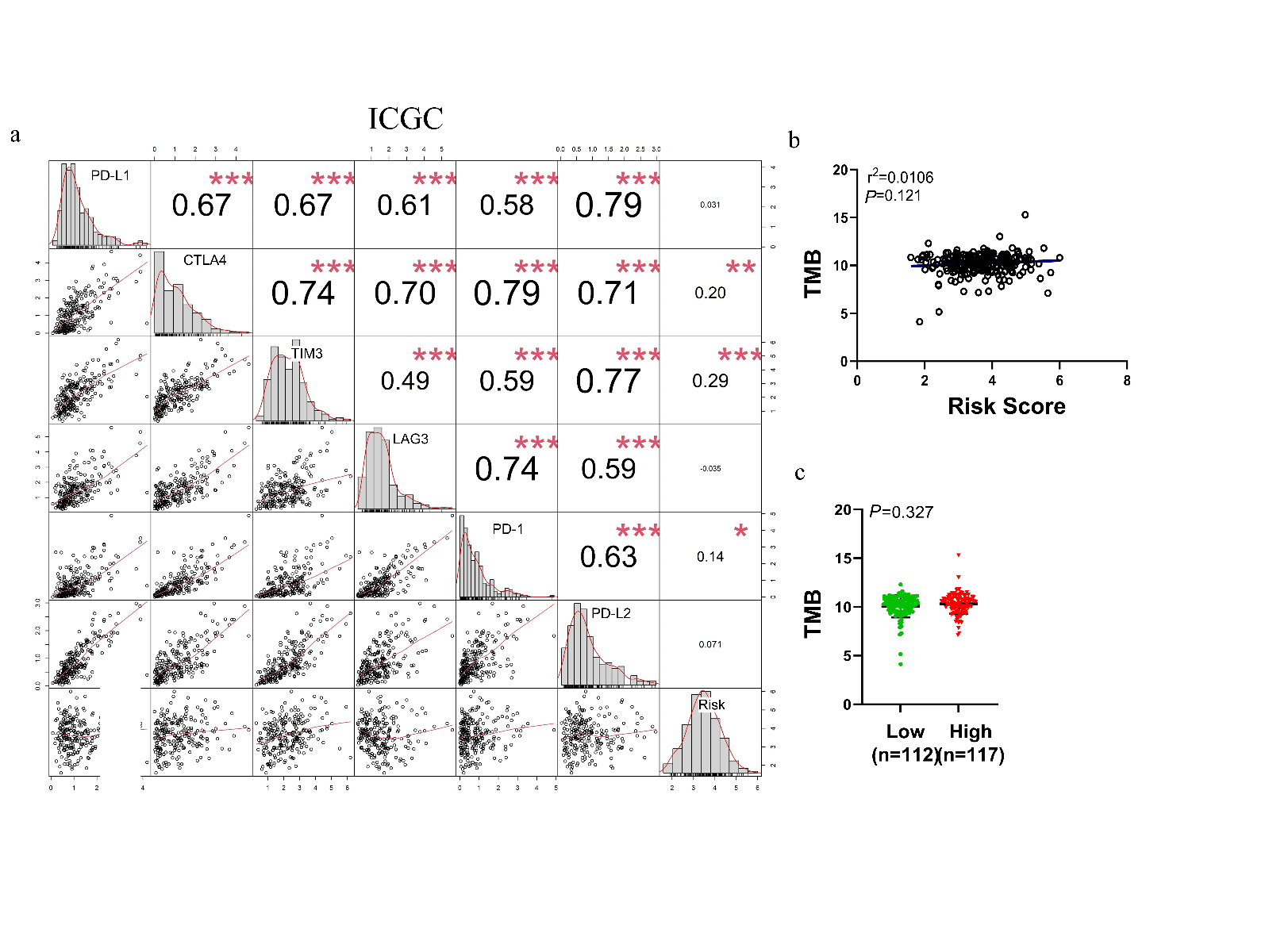


Fig. S15 Relationship between the risk signature and immune checkpoints. (a) Correlation between the risk scores and the expression of PD-L1, CTLA-4, TIM3, LAG3, PD-1 and PD-L2 in the ICGC cohort (Pearson correlation analysis). (b, c) Correlation between the risk score and the expression of TMB in the IGGC cohort (Pearson correlation analysis) in each patient (b) and low- and high- groups (c).


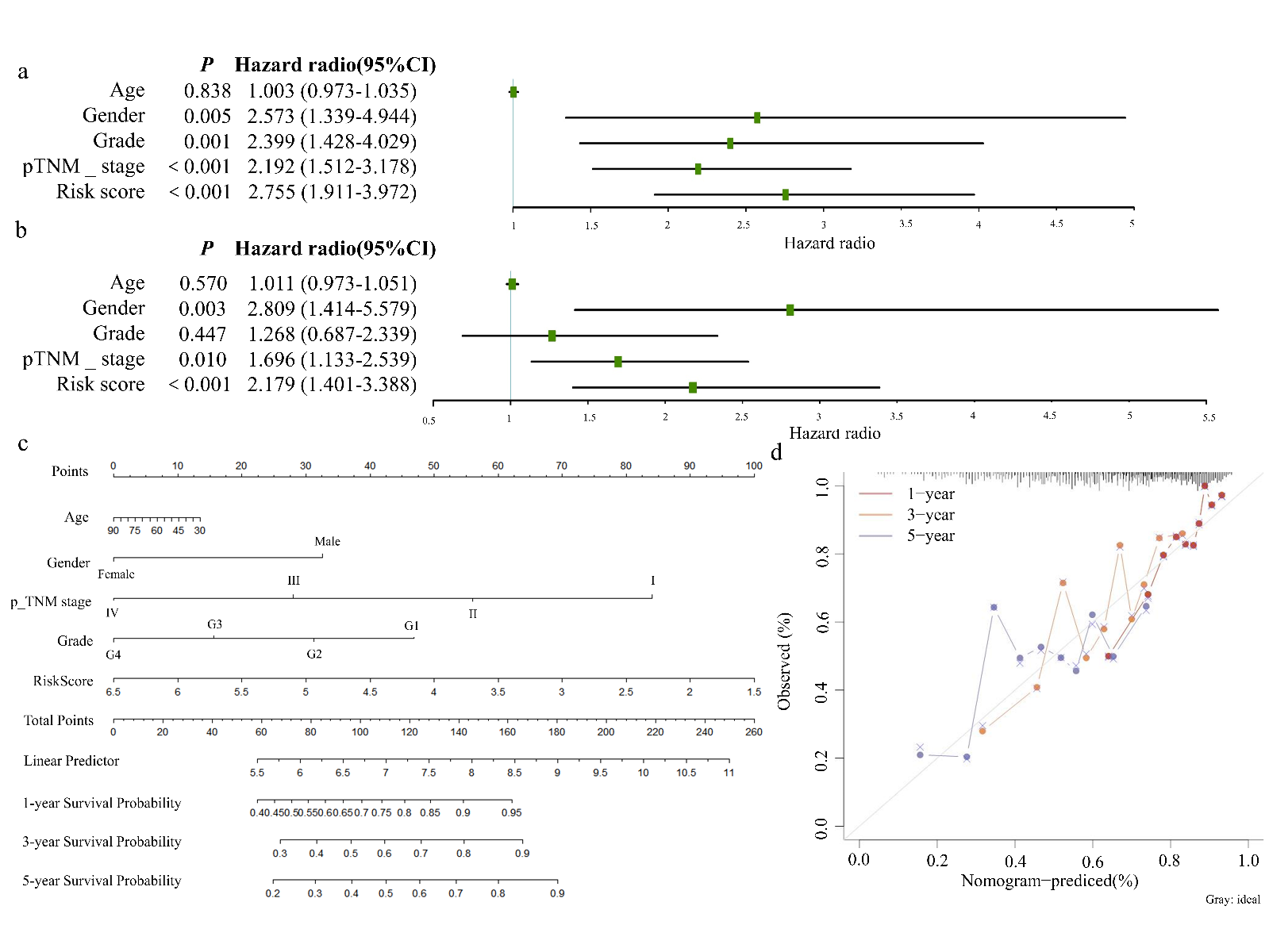


Fig. S16 Forest plot of the univariate (a) and multivariate (b) Cox regression analysis in the ICGC cohorts. (c, d) The nomogram (c) and the calibration curve (d) analyses were performed to predict 1-, 3-, and 5-year OS according to risk score.

| Table S1 124 factors were identified as lipid droplet-associated factors by literature search. | | | | | | | | | |
| --- | --- | --- | --- | --- | --- | --- | --- | --- | --- |
| Factors |  | Factors |  | Factors |  | Factors |  | Factors |  |
| ABHD5 | [1][2][3] | CIDEB | [29] | GIMAP2 | [51] | NNMT | [76] | RBP1 | [105] |
| ACAT1 | [2] | CIDEC | [29] [30] | GPAT4 | [52] | NSDHL | [77] | RSAD2 | [106] |
| ACSL3 | [4] | CKAP4 | [31] | HILPDA | [53][54][55] | NSF | [78] | SCCPDH | [107] |
| ACSL4 | [5] | CPT1A | [7] | HSD17B11 | [56] | OSBPL2 | [79] | SCD | [44][53] |
| ACOX1 | [6][7] | CTDNEP1 | [32] | HSD17B13 | [57][58] | PCYT1A | [80] | SET | [18] |
| AGPAT2 | [8] | CYB5R3 | [33] | HSD3B7 | [18] | PEMT | [81][82] | SETSIP | [18] |
| AIFM2 | [9] | DBC1 | [34] | HSPA5 | [21] | PITPNM1 | [83] | SIGMAR1 | [108] |
| AMPK | [6] | DFFA | [18] | ICE2 | [59] | PLD1 | [84][85] | SNAP23 | [109] |
| ANGPTL8 | [10] | DGAT1 | [2][35] | IRAK1 | [60] | PLIN1 | [1][73][7][86] | SPAST | [110] |
| ANXA2 | [11] | DGAT2 | [36][37] | LDAH | [61] | PLIN2 | [14][26][73][86][15][30][55][87][88][89] | SPG20 | [111] |
| APOA4 | [12] | DNAAF1 | [30] | LIPE | [1][11] | PLIN3 | [90][73][86][91][92][87] | SQLE | [112] |
| APOB | [13] | EDA | [38] | LMLN | [62] | PLIN4 | [55][73][93][44] | STARD13 | [113] |
| AQP1 | [14][15] | EHD1 | [39] | LPCAT1 | [63][64][65] | PLIN5 | [73] | STX5 | [109] |
| ARAP2 | [16] | FAAH2 | [40] | LPCAT2 | [66] | PNPLA2 | [1][94] | SYNGR2 | [115] |
| ATG2A | [17] | FABP1 | [41] | LPIN1 | [67] | PNPLA3 | [58][95] | THRSP | [44] |
| ATG2B | [18][19] | FABP4 | [7] | LSS | [68] | PNPLA4 | [95] | TMEM135 | [58] |
| AUP1 | [20][21] | FAF2 | [42][43] | MAP4K4 | [69] | PNPLA5 | [96] | TPD52 | [115] |
| BCAP31 | [18] | FASN | [7] [44] | MBOAT7 | [58] | PRPF19 | [97] | TRAF6 | [116] |
| BSCL2 | [22] | FGF21 | [45] | METTL7A | [70] | RAB18 | [90][98][99] | TSC1 | [117] |
| CAV1 | [18][23] | FIG4 | [18] | METTL7B | [70][71] | RAB3GAP1 | [100] | UBE2G2 | [21] |
| CAV2 | [24] | FITM1 | [46] | MGLL | [72] | RAB5A | [101][56][102] | VAMP4 | [109] |
| CAVIN1 | [25] | FITM2 | [46] | MLDP | [73] | RAB5C | [101][56][102] | VAPA | [79] |
| CDKN1A | [26] | G0S2 | [47][48] | MTTP | [74] | RAB7A | [101][56][102] | VCP | [118] |
| CES1 | [27] | GAPDH | [49] | NAPA | [42] | RAB8A | [103] | VMP1 | [119] [120] |
| CIDEA | [28][29] | GBF1 | [50] | NCEH1 | [75] | RAP1B | [104] |  |  |
| ABHD5: Abhydrolase 5  ACAT1: Acetyl-CoA acetyltransferase 1  ACSL3, ACSL4: Acyl-CoA synthetase long chain family member 3, 4  ACOX1: Acyl-CoA oxidase 1  AGPAT2: 1-acylglycerol-3-phosphate O-acyltransferase 2  AIFM2: Apoptosis-inducing factor mitochondria-associated 2  AMPK: AMP-activated protein kinase  ANGPTL8: Angiopoietin like 8  ANXA2: Annexin A2  APOA4, APOB: Apolipoprotein A4, B  AQP1: Aquaporin 1  ARAP2: ADP-ribosylation factor GTPase-activating protein  ATG2A, ATG2B: Autophagy-related gene  AUP1: Ancient ubiquitous protein 1  BCAP31: B cell receptor-associated protein 31  BSCL2: Berardinelli-Seip congenital lipodystrophy 2  CAV1, CAV2: Caveolin 1, 2  CAVIN1: Caveolae-associated protein 1  CDKN1A: Cyclin-dependent kinase inhibitor 1A  CES1: Carboxyesterase 1  CIDEA, CIDEB, CIDEC: Cell death-inducing DNA fragmentation factor-like effector family members A, B, C  CKAP4: Cytoskeleton-associated protein 4  CPT1A: Carnitine palmitoyltransferase 1A  CTDNEP1: CTD Nuclear Envelope Phosphatase 1  CYB5R3: Cytochrome b5 reductase 3  DBC1: Deleted in Breast Cancer 1  DFFA: DNA Fragmentation Factor Alpha  DGAT1, DGAT2: Diacylglycerol O-Acyltransferase  DNAAF1: Dynein axonemal assembly factor 1  EDA: Ectodysplasin A  EHD1: Eps15 homology domain-containing 1  FAAH2: Fatty acid amide hydrolase 2  FABP1, FABP4: Fatty acid binding protein  FAF2: Fas-associated factor 2  FASN: Fatty acid synthase  FGF21: Fibroblast growth factor 21  FIG4: Phosphoinositide 5-Phosphatase  FITM1, FITM2: Fat storage inducing transmembrane Protein  G0S2: G0/G1 switch protein  GAPDH: Glyceraldehyde-3-phosphate dehydrogenase  GBF1: Golgi brefeldin A resistance factor 1  GIMAP2: GTPases of immunity-associated protein 2  GPAT4: Glycerol-3-phosphate acyltransferase 4  HILPDA: Hypoxia inducible LD associated  HSD17B11, HSD17B13: Hydroxysteroid 11, 13  HSD3B7: 3beta-hydroxy-delta(5)-C(27)-steroid oxidoreductase  HSPA5: Heat shock protein A5  ICE2: Interactor of little elongation complex ELL 2  IRAK1: Interleukin-1 receptor-associated kinase  LDAH: LD-associated hydrolase  LIPE: Lipase E  LMLN: Leishmanolysin Like Peptidase  LPCAT1, LPCAT2: Lysophosphatidylcholine Acyltransferase  LSS: Lanosterol synthase  MAP4K4: Mitogen-Activated Protein Kinase Kinase Kinase Kinase 4  MBOAT7: Membrane Bound O-Acyltransferase Domain Containing 7  METTL7A, METTL7B: Methyltransferase like 7A, 7B  MGLL: Monoglyceride lipase  MLDP: Myocardial LD protein  MTTP: Microsomal triglyceride transfer protein  NAPA: N-Ethylmaleimide-sensitive factor-attachment protein α  NCEH1: Neutral cholesterolester hydrolase 1  NNMT: Nicotinamide N-Methyltransferase  NSDHL: NAD(P) Dependent Steroid Dehydrogenase-Like  NSF: Ethylmaleimide-sensitive factor  OSBPL2: Oxysterol binding protein like 2  PCYT1A: Phosphate Cytidylyl transferase 1A  PEMT: Phosphatidylethanolamine N-methytransferase  PITPNM1: Phosphatidylinositol Transfer Protein Membrane Associated 1  PLD1: Phospholipase 1  PNPLA2, PNPLA3, PNPLA4, PNPLA5: Patatin-like phospholipase domain containing A2, A3, A4, A5  PRPF19: Pre-mRNA Processing Factor 19  RAB3GAP1: RAB3 GTPase Activating Protein Catalytic Subunit 1  RAB18, RAB5A, RAB5C, RAB7A, RAB8A: RAS oncogene family 18, 5A, 5C, 7A, 8A, P1B  RBP1: Retinol Binding Protein 1  RSAD2: Radical S-Adenosyl Methionine Domain Containing 2  SCCPDH: Saccharopine Dehydrogenase  SCD: Stearoyl-CoA Desaturase  SET: Nuclear Proto-Oncogene  SETSIP: SET Like Protein  SIGMAR1: Sigma Non-Opioid Intracellular Receptor 1  SNAP23: Synaptosome Associated Protein 23  SPAST: Spastin  SPG20: Spastic Paraplegia 20  SQLE: Squalene Epoxidase  STARD13: StAR Related Lipid Transfer Domain Containing 13  STX5: Synthaxin 5  SYNGR2: Synaptogyrin 2  THRSP: Thyroid Hormone Responsive  TMEM135: Transmembrane Protein 135  TPD52: Tumor Protein D52  TRAF6: TNF Receptor Associated Factor 6  TSC1: TSC Complex Subunit 1  UBE2G2: Ubiquitin Conjugating Enzyme E2 G2  VAMP4: Vesicle associated membrane protein 4  VAPA: Vesicle-associated membrane protein-associated protein  VMP1: Vesicular membrane Protein 1 | | | | | | | | | |

**Reference**

1. Silvério R, Lira FS, Oyama LM, et al. Lipases and lipid droplet-associated protein expression in subcutaneous white adipose tissue of cachectic patients with cancer. Lipids Health Dis. 2017, 16(1):159.

2. Mitra R, Le TT, Gorjala P, et al. Positive regulation of prostate cancer cell growth by lipid droplet forming and processing enzymes DGAT1 and ABHD5. BMC Cancer. 2017, 17(1):631.

3. Eichmann TO, Grumet L, Taschler U, et al. ATGL and CGI-58 are lipid droplet proteins of the hepatic stellate cell line HSC-T6. J Lipid Res. 2015, 56(10):1972-1984.

4. Migita T, Takayama KI, Urano T, et al. ACSL3 promotes intratumoral steroidogenesis in prostate cancer cells. Cancer Sci. 2017, 108(10):2011-2021.

5. Zimmermann R, Strauss JG, Haemmerle G, et al. Fat mobilization in adipose tissue is promoted by adipose triglyceride lipase. Science. 2004, 306(5700):1383-1386.

6. Zang Yufan, Fan Li, Chen Jihua, et al. Improvement of Lipid and Glucose Metabolism by Capsiate in Palmitic Acid-Treated HepG2 Cells via Activation of the AMPK/SIRT1 Signaling Pathway. J Agric Food Chem. 2018, 66(26):6772-6781.

7. Jung YY, Kim HM, Koo JS. Expression of Lipid Metabolism-Related Proteins in Metastatic Breast Cancer. PLoS One. 2015, 10(9):e0137204.

8. González-Hódar L, McDonald JG, Vale G, et al. Decreased caveolae in AGPAT2 lacking adipocytes is independent of changes in cholesterol or sphingolipid levels: A whole cell and plasma membrane lipidomic analysis of adipogenesis. Biochim Biophys Acta Mol Basis Dis. 2021, 1867(9):166167.

9. Nguyen HP, Yi D, Lin F, et al. Aifm2, a NADH Oxidase, Supports Robust Glycolysis and Is Required for Cold- and Diet-Induced Thermogenesis. Mol Cell. 2020, 77(3):600-617.

10. Tseng YH, Ke PY, Liao CJ, et al. Chromosome 19 open reading frame 80 is upregulated by thyroid hormone and modulates autophagy and lipid metabolism. Autophagy. 2014, 10(1):20-31.

11. Drücker P, Pejic M, Galla HJ, et al. Lipid segregation and membrane budding induced by the peripheral membrane binding protein annexin A2. J Biol Chem. 2013, 288(34):24764-24776.

12. Qu J, Fourman S, Fitzgerald M, et al. Low-density lipoprotein receptor-related protein 1 (LRP1) is a novel receptor for apolipoprotein A4 (APOA4) in adipose tissue. Sci Rep. 2021, 11(1):13289.

13. Walsh MT, Celestin OM, Thierer JH, et al. Model systems for studying the assembly, trafficking, and secretion of apoB lipoproteins using fluorescent fusion proteins. J Lipid Res. 2020, 61(3):316-327.

14. Morrissey JJ, Mobley J, Figenshau RS, et al. Urine aquaporin 1 and perilipin 2 differentiate renal carcinomas from other imaged renal masses and bladder and prostate cancer. Mayo Clin Proc. 2015, 90(1):35-42.

15. Morrissey JJ, Mellnick VM, Luo J, et al. Evaluation of Urine Aquaporin-1 and Perilipin-2 Concentrations as Biomarkers to Screen for Renal Cell Carcinoma: A Prospective Cohort Study. JAMA Oncol. 2015, 1(2):204-212.

16. Chaudhari A, Håversen L, Mobini R, et al. ARAP2 promotes GLUT1-mediated basal glucose uptake through regulation of sphingolipid metabolism. Biochim Biophys Acta. 2016, 1861(11):1643-1651.

17. Valverde DP, Yu Shenliang, Boggavarapu V, et al. ATG2 transports lipids to promote autophagosome biogenesis. J Cell Biol. 2019, 218(6):1787-1798.

18. Choi K, Jin M, Zouboulis CC, et al. Increased Lipid Accumulation under Hypoxia in SZ95 Human Sebocytes. Dermatology. 2021, 237(1):131-141.

19. Osawa T, Ishii Y, Noda NN. Human ATG2B possesses a lipid transfer activity which is accelerated by negatively charged lipids and WIPI4. Genes Cells. 2020, 25(1):65-70.

20. Jo Y, Hartman IZ, DeBose-Boyd RA. Ancient ubiquitous protein-1 mediates sterol-induced ubiquitination of 3-hydroxy-3-methylglutaryl CoA reductase in lipid droplet-associated endoplasmic reticulum membranes. Mol Biol Cell. 2013, 24(3):169-183.

21. Robichaud S, Fairman G, Vijithakumar V, et al. Identification of novel lipid droplet factors that regulate lipophagy and cholesterol efflux in macrophage foam cells. Autophagy. 2021, 17(11):3671-3689.

22. Mcilroy GD, Mitchell SE, Han Weiping, et al. Ablation of *Bscl2*/seipin in hepatocytes does not cause metabolic dysfunction in congenital generalised lipodystrophy. Dis Model Mech. 2020, 13(1):dmm042655.

23. Kim CA, Delépine M, Boutet E, et al. Association of a homozygous nonsense caveolin-1 mutation with Berardinelli-Seip congenital lipodystrophy. J Clin Endocrinol Metab. 2008, 93(4):1129-1134.

24. Cohen R, Buttke DE, Asano A, et al. Lipid modulation of calcium flux through CaV2.3 regulates acrosome exocytosis and fertilization. Dev Cell. 2014 Feb 10;28(3):310-21.

25. Wei Zhuang, Lei Jigang, Shen Feng, et al. Cavin1 Deficiency Causes Disorder of Hepatic Glycogen Metabolism and Neonatal Death by Impacting Fenestrations in Liver Sinusoidal Endothelial Cells. Adv Sci (Weinh). 2020, 7(19):2000963.

26. Chen Xue, Wang Kesheng, Cederbaum AI, et al. Suppressed hepatocyte proliferation via a ROS-HNE-P21 pathway is associated with nicotine- and cotinine-enhanced alcoholic fatty liver in mice. Biochem Biophys Res Commun. 2019, 512(1):119-124.

27. Zhou Lijie, Zhang Cai, Yang Xiong, et al. Melatonin inhibits lipid accumulation to repress prostate cancer progression by mediating the epigenetic modification of CES1. Clin Transl Med. 2021, 11(6):e449.

28. Son Y, Choi C, Song C, et al. Development of CIDEA reporter mouse model and its application for screening thermogenic drugs. Sci Rep. 2021, 11(1):18429.

29. Yu Ming, Wang Hui, Zhao Jun, et al. Expression of CIDE proteins in clear cell renal cell carcinoma and their prognostic significance. Mol Cell Biochem. 2013, 378(1-2):145-51.

30. Liu Zhichao, Wang Yuping, Borlak J, et al. Mechanistically linked serum miRNAs distinguish between drug induced and fatty liver disease of different grades. Sci Rep. 2016, 6:23709.

31. Sabbisetti V, Di Napoli A, Seeley A, et al. p63 promotes cell survival through fatty acid synthase. PLoS One. 2009, 4(6):e5877.

32. Jacquemyn J, Foroozandeh J, Vints K, et al. Torsin and NEP1R1-CTDNEP1 phosphatase affect interphase nuclear pore complex insertion by lipid-dependent and lipid-independent mechanisms. EMBO J. 2021, 40(17):e106914.

33. Wei Lisha, Zheng Yanyan, Sun Jie, et al. GGPP depletion initiates metaflammation through disequilibrating CYB5R3-dependent eicosanoid metabolism. J Biol Chem. 2020, 295(47):15988-16001.

34. Moreno-Navarrete JM, Moreno M, Vidal M, et al. Deleted in breast cancer 1 plays a functional role in adipocyte differentiation. Am J Physiol Endocrinol Metab. 2015, 308(7):E554-61.

35. de la Rosa Rodriguez MA, Deng Lei, Gemmink A, et al. Hypoxia-inducible lipid droplet-associated induces DGAT1 and promotes lipid storage in hepatocytes. Mol Metab. 2021, 47:101168.

36. Kuerschner L, Moessinger C, Thiele C. Imaging of lipid biosynthesis: how a neutral lipid enters lipid droplets. Traffic. 2008, 9(3):338-352.

37. Stone SJ, Levin MC, Zhou Ping, et al. The endoplasmic reticulum enzyme DGAT2 is found in mitochondria-associated membranes and has a mitochondrial targeting signal that promotes its association with mitochondria. J Biol Chem. 2009, 284(8):5352-5361.

38. Stürner KH, Werz O, Koeberle A, et al. Lipid Mediator Profiles Predict Response to Therapy with an Oral Frankincense Extract in Relapsing-Remitting Multiple Sclerosis. Sci Rep. 2020, 10(1):8776.

39. Naslavsky N, Rahajeng J, Rapaport D, et al. EHD1 regulates cholesterol homeostasis and lipid droplet storage. Biochem Biophys Res Commun. 2007, 357(3):792-799.

40. Kaczocha M, Glaser ST, Chae J, et al. Lipid droplets are novel sites of N-acylethanolamine inactivation by fatty acid amide hydrolase-2. J Biol Chem. 2010, 285(4):2796-2806.

41. Wang Yunqiu, Jia Mengqi, Liang Chuanjie, et al. Anterior gradient 2 increases long-chain fatty acid uptake via stabilizing FABP1 and facilitates lipid accumulation. Int J Biol Sci. 2021, 17(3):834-847.

42. Landreh M, Costeira-Paulo J, Gault J, et al. Effects of Detergent Micelles on Lipid Binding to Proteins in Electrospray Ionization Mass Spectrometry. Anal Chem. 2017, 89(14):7425-7430.

43. Suzuki M, Otsuka T, Ohsaki Y, et al. Derlin-1 and UBXD8 are engaged in dislocation and degradation of lipidated ApoB-100 at lipid droplets. Mol Biol Cell. 2012, 23(5):800-810.

44. Anthérieu S, Rogue A, Fromenty B, et al. Induction of vesicular steatosis by amiodarone and tetracycline is associated with up-regulation of lipogenic genes in HepaRG cells. Hepatology. 2011, 53(6):1895-1905.

45. Arner P, Pettersson A, Mitchell PJ, et al. FGF21 attenuates lipolysis in human adipocytes - a possible link to improved insulin sensitivity. FEBS Lett. 2008, 582(12):1725-1730.

46. Kadereit B, Kumar P, Wang Wenjun, et al. Evolutionarily conserved gene family important for fat storage. Proc Natl Acad Sci U S A. 2008, 105(1):94-99.

47. Zandbergen F, Mandard S, Escher P, et al. The G0/G1 switch gene 2 is a novel PPAR target gene. Biochem J. 2005, 392(Pt 2):313-24.

48. Yang Xingyuan, Lu Xin, Lombès M, et al. The G(0)/G(1) switch gene 2 regulates adipose lipolysis through association with adipose triglyceride lipase. Cell Metab. 2010, 11(3):194-205.

49. Gouzy A, Healy C, Black KA, et al. Growth of *Mycobacterium tuberculosis* at acidic pH depends on lipid assimilation and is accompanied by reduced GAPDH activity. Proc Natl Acad Sci U S A. 2021, 118(32):e2024571118.

50. Bouvet S, Golinelli-Cohen MP, Contremoulins V, et al. Targeting of the Arf-GEF GBF1 to lipid droplets and Golgi membranes. J Cell Sci. 2013, 126(Pt 20):4794-805.

51. Schwefel D, Fröhlich C, Eichhorst J, et al. Structural basis of oligomerization in septin-like GTPase of immunity-associated protein 2 (GIMAP2). Proc Natl Acad Sci U S A. 2010, 107(47):20299-20304.

52. Wilfling F, Wang Huajin, Haas JT, et al. Triacylglycerol synthesis enzymes mediate lipid droplet growth by relocalizing from the ER to lipid droplets. Dev Cell. 2013, 24(4):384-399.

53. Schübel R, Sookthai D, Greimel J, et al. Key Genes of Lipid Metabolism and WNT-Signaling Are Downregulated in Subcutaneous Adipose Tissue with Moderate Weight Loss. Nutrients. 2019, 11(3):639.

54. Sheng Yanrui, Li Jinlong, Yang Yanna, et al. Hypoxia-inducible lipid droplet-associated (HILPDA) facilitates the malignant phenotype of lung adenocarcinoma cells in vitro through modulating cell cycle pathways. Tissue Cell. 2021, 70:101495.

55. Schley G, Grampp S, Goppelt-Struebe M. Inhibition of oxygen-sensing prolyl hydroxylases increases lipid accumulation in human primary tubular epithelial cells without inducing ER stress. Cell Tissue Res. 2020, 381(1):125-140.

56. Fujimoto Y, Itabe H, Sakai J, et al. Identification of major proteins in the lipid droplet-enriched fraction isolated from the human hepatocyte cell line HuH7. Biochim Biophys Acta. 2004 Feb 2;1644(1):47-59.

57. Horiguchi Y, Araki M, Motojima K. 17beta-Hydroxysteroid dehydrogenase type 13 is a liver-specific lipid droplet-associated protein. Biochem Biophys Res Commun. 2008, 370(2):235-8.

58. Landowski M, Bhute VJ, Takimoto T, et al. A mutation in transmembrane protein 135 impairs lipid metabolism in mouse eyecups. Sci Rep. 2022, 12(1):756.

59. Markgraf DF, Klemm RW, Junker M, et al. An ER protein functionally couples neutral lipid metabolism on lipid droplets to membrane lipid synthesis in the ER. Cell Rep. 2014, 6(1):44-55.

60. Lei Ying, Yang Tianxiao, Shan Aijing, et al. Altered Inflammatory Pathway but Unaffected Liver Fibrosis in Mouse Models of Nonalcoholic Steatohepatitis Involving Interleukin-1 Receptor-Associated Kinase 1 Knockout. Med Sci Monit. 2020, 26:e926187.

61. Goo YH, Son SH, Kreienberg PB, et al. Novel lipid droplet-associated serine hydrolase regulates macrophage cholesterol mobilization. Arterioscler Thromb Vasc Biol. 2014, 34(2):386-396.

62. Vass S, Heck MM. Perturbation of invadolysin disrupts cell migration in zebrafish (Danio rerio). Exp Cell Res. 2013, 319(8):1198-1212.

63. Abdelzaher E, Mostafa MF. Lysophosphatidylcholine acyltransferase 1 (LPCAT1) upregulation in breast carcinoma contributes to tumor progression and predicts early tumor recurrence. Tumour Biol. 2015, 36(7):5473-5483.

64. Mansilla F, da Costa KA, Wang Shuli, et al. Lysophosphatidylcholine acyltransferase 1 (LPCAT1) overexpression in human colorectal cancer. J Mol Med (Berl). 2009, 87(1):85-97.

65. Zhou Xinchun, Lawrence TJ, He Zhi, et al. The expression level of lysophosphatidylcholine acyltransferase 1 (LPCAT1) correlates to the progression of prostate cancer. Exp Mol Pathol. 2012, 92(1):105-110.

66. Williams KA, Lee M, Hu Ying, et al. A systems genetics approach identifies CXCL14, ITGAX, and LPCAT2 as novel aggressive prostate cancer susceptibility genes. PLoS Genet. 2014, 10(11):e1004809.

67. Romani P, Brian I, Santinon G, et al. Extracellular matrix mechanical cues regulate lipid metabolism through Lipin-1 and SREBP. Nat Cell Biol. 2019, 21(3):338-347.

68. Cohain AT, Barrington WT, Jordan DM, et al. An integrative multiomic network model links lipid metabolism to glucose regulation in coronary artery disease. Nat Commun. 2021, 12(1):547.

69. Roth Flach RJ, DiStefano MT, Danai LV, et al. Map4k4 impairs energy metabolism in endothelial cells and promotes insulin resistance in obesity. Am J Physiol Endocrinol Metab. 2017, 313(3):E303-E313.

70. Zehmer JK, Bartz R, Liu Pingsheng, et al. Identification of a novel N-terminal hydrophobic sequence that targets proteins to lipid droplets. J Cell Sci. 2008, 121(11):1852-1860.

71. Turró S, Ingelmo-Torres M, Estanyol JM, et al. Identification and characterization of associated with lipid droplet protein 1: A novel membrane-associated protein that resides on hepatic lipid droplets. Traffic. 2006, 7(9):1254-1269.

72. Xiang Wei, Shi Rongchen, Kang Xia, et al. Monoacylglycerol lipase regulates cannabinoid receptor 2-dependent macrophage activation and cancer progression. Nat Commun. 2018 Jul 3;9(1):2574.

73. Pawella LM, Hashani M, Schirmacher P, et al. Effekte einer Induktion und siRNA-vermittelten Herunterregulation von PAT-Proteinen in hepatozytären Zellkulturmodellen [Lipid droplet-associated proteins in steatosis. Effects of induction and siRNA-mediated downregulation of PAT proteins in cell culture models of hepatocyte steatosis]. Pathologe. 2010, 31 Suppl 2:126-131.

74. Grünig D, Szabo L, Marbet M, et al. Valproic acid affects fatty acid and triglyceride metabolism in HepaRG cells exposed to fatty acids by different mechanisms. Biochem Pharmacol. 2020, 177:113860.

75. Lu Yan, Zhang Longyi, Chen Xuya, et al. NCEH1 may be a prognostic biomarker for pancreatic cancer. Int J Clin Exp Pathol. 2020, 13(11):2746-2752.

76. Komatsu M, Kanda T, Urai H, et al. NNMT activation can contribute to the development of fatty liver disease by modulating the NAD ^+^ metabolism. Sci Rep. 2018, 8(1):8637.

77. Zhang Haiyan, Li Chengping, Xin Youzhi, et al. Suppression of *NSDHL* attenuates adipogenesis with a downregulation of LXR-SREBP1 pathway in 3T3-L1 cells. Biosci Biotechnol Biochem. 2020, 84(5):980-988.

78. Starr ML, Fratti R. Determination of Sec18-Lipid Interactions by Liposome-Binding Assay. Methods Mol Biol. 2019, 1860:211-220.

79. Weber-Boyvat M, Kentala H, Peränen J, et al. Ligand-dependent localization and function of ORP-VAP complexes at membrane contact sites. Cell Mol Life Sci. 2015, 72(10):1967-1987.

80. Andrejeva G, Gowan S, Lin Gigin, et al. *De novo* phosphatidylcholine synthesis is required for autophagosome membrane formation and maintenance during autophagy. Autophagy. 2020, 16(6):1044-1060.

81. Fei Weihua, Shui Guanghou, Gaeta B, et al. Fld1p, a functional homologue of human seipin, regulates the size of lipid droplets in yeast. J Cell Biol. 2008, 180(3):473-482.

82. Wang Chaowen, Miao YH, Chang Yishu. Control of lipid droplet size in budding yeast requires the collaboration between Fld1 and Ldb16. J Cell Sci. 2014, 127(Pt 6):1214-1228.

83. Litvak V, Shaul YD, Shulewitz M, et al. Targeting of Nir2 to lipid droplets is regulated by a specific threonine residue within its PI-transfer domain. Curr Biol. 2002, 12(17):1513-1518.

84. Marchesan D, Rutberg M, Andersson L, et al. A phospholipase D-dependent process forms lipid droplets containing caveolin, adipocyte differentiation-related protein, and vimentin in a cell-free system. J Biol Chem. 2003, 278(29):27293-27300.

85. Andersson L, Boström P, Ericson J, et al. PLD1 and ERK2 regulate cytosolic lipid droplet formation. J Cell Sci. 2006, 119(Pt 11):2246-2257.

86. Straub BK, Herpel E, Singer S, et al. Lipid droplet-associated PAT-proteins show frequent and differential expression in neoplastic steatogenesis. Mod Pathol. 2010, 23(3):480-492.

87. Kaushik S, Cuervo AM. Degradation of lipid droplet-associated proteins by chaperone-mediated autophagy facilitates lipolysis. Nat Cell Biol. 2015, 17(6):759-770.

88. Tsai TH, Chen E, Li Lan, et al. The constitutive lipid droplet protein PLIN2 regulates autophagy in liver. Autophagy. 2017, 13(7):1130-1144.

89. Kuniyoshi S, Miki Y, Sasaki A, et al. The significance of lipid accumulation in breast carcinoma cells through perilipin 2 and its clinicopathological significance. Pathol Int. 2019, 69(8):463-471.

90. Ohsaki Y, Maeda T, Fujimoto T. Fixation and permeabilization protocol is critical for the immunolabeling of lipid droplet proteins. Histochem Cell Biol. 2005, 124(5):445-452.

91. Yao Dayong, Xia Shunyao, Jin Chengjun, et al. Feedback activation of GATA1/miR-885-5p/PLIN3 pathway decreases sunitinib sensitivity in clear cell renal cell carcinoma. Cell Cycle. 2020, 19(17):2195-2206.

92. El-Ekiaby NM, Mekky RY, El Sobky SA, et al. Epigenetic harnessing of HCV via modulating the lipid droplet-protein, TIP47, in HCV cell models. FEBS Lett. 2015, 589(17):2266-2273.

93. Sirois I, Aguilar-Mahecha A, Lafleur J, et al. A Unique Morphological Phenotype in Chemoresistant Triple-Negative Breast Cancer Reveals Metabolic Reprogramming and PLIN4 Expression as a Molecular Vulnerability. Mol Cancer Res. 2019, 17(12):2492-2507.

94. Grönke S, Mildner A, Fellert S, et al. Brummer lipase is an evolutionary conserved fat storage regulator in Drosophila. Cell Metab. 2005, 1(5):323-330.

95. Chen Zhilong, Gao Xin, Lei Ting, et al. Molecular characterization, expression and chromosomal localization of porcine PNPLA3 and PNPLA4. Biotechnol Lett. 2011, 33(7):1327-1337.

96. Dupont N, Chauhan S, Arko-Mensah J, et al. Neutral lipid stores and lipase PNPLA5 contribute to autophagosome biogenesis. Curr Biol. 2014, 24(6):609-620.

97. Cho SY, Shin ES, Park PJ, et al. Identification of mouse Prp19p as a lipid droplet-associated protein and its possible involvement in the biogenesis of lipid droplets. J Biol Chem. 2007, 282(4):2456-2465.

98. Ozeki S, Cheng Jinglei, Tauchi-Sato K, et al. Rab18 localizes to lipid droplets and induces their close apposition to the endoplasmic reticulum-derived membrane. J Cell Sci. 2005, 118(Pt 12):2601-2611.

99. Martin S, Driessen K, Nixon SJ, et al. Regulated localization of Rab18 to lipid droplets: effects of lipolytic stimulation and inhibition of lipid droplet catabolism. J Biol Chem. 2005, 280(51):42325-42335.

100. Spang N, Feldmann A, Huesmann H, et al. RAB3GAP1 and RAB3GAP2 modulate basal and rapamycin-induced autophagy. Autophagy. 2014, 10(12):2297-2309.

101. Liu Pingsheng, Ying Yunshu, Zhao Yingming, et al. Chinese hamster ovary K2 cell lipid droplets appear to be metabolic organelles involved in membrane traffic. J Biol Chem. 2004, 279(5):3787-3792.

102. Bartz R, Zehmer JK, Zhu M, et al. Dynamic activity of lipid droplets: protein phosphorylation and GTP-mediated protein translocation. J Proteome Res. 2007, 6(8):3256-3265.

103. Wu Lizhen, Xu Dijin, Zhou Linkang, et al. Rab8a-AS160-MSS4 regulatory circuit controls lipid droplet fusion and growth. Dev Cell. 2014, 30(4):378-393.

104. Kumar S, Xu Juying, Kumar RS, et al. The small GTPase Rap1b negatively regulates neutrophil chemotaxis and transcellular diapedesis by inhibiting Akt activation. J Exp Med. 2014, 211(9):1741-1758.

105. Bennick RA, Nagengast AA, DiAngelo JR. The SR proteins SF2 and RBP1 regulate triglyceride storage in the fat body of Drosophila. Biochem Biophys Res Commun. 2019, 516(3):928-933.

106. Jang Jisu, Lee JH, Jung NC, et al. Rsad2 is necessary for mouse dendritic cell maturation via the IRF7-mediated signaling pathway. Cell Death Dis. 2018, 9(8):823.

107. Dakik H, Mantash S, Nehme A, et al. Analysis of the Neuroproteome Associated With Cell Therapy After Intranigral Grafting in a Mouse Model of Parkinson Disease. Front Neurosci. 2021, 15:621121.

108. Gueguinou M, Crottès D, Chantôme A, et al. The SigmaR1 chaperone drives breast and colorectal cancer cell migration by tuning SK3-dependent Ca^2+^ homeostasis. Oncogene. 2017, 36(25):3640-3647.

109. Boström P, Andersson L, Rutberg M, et al. SNARE proteins mediate fusion between cytosolic lipid droplets and are implicated in insulin sensitivity. Nat Cell Biol. 2007, 9(11):1286-1293.

110. Papadopoulos C, Orso G, Mancuso G, et al. Spastin binds to lipid droplets and affects lipid metabolism. PLoS Genet. 2015, 11(4):e1005149.

111. Eastman SW, Yassaee M, Bieniasz PD. A role for ubiquitin ligases and Spartin/SPG20 in lipid droplet turnover. J Cell Biol. 2009, 184(6):881-94.

112. Liu Dabin, Wong Chichun, Zhou Yunfei, et al. Squalene Epoxidase Induces Nonalcoholic Steatohepatitis Via Binding to Carbonic Anhydrase III and is a Therapeutic Target. Gastroenterology. 2021, 160(7):2467-2482.

113. Chen Lin, Hu Weifeng, Li Guohao, et al. Inhibition of miR-9-5p suppresses prostate cancer progress by targeting StarD13. Cell Mol Biol Lett. 2019, 24:20.

114. Sun Qiyun, Qi Xian, Zhang Yan, et al. Synaptogyrin-2 Promotes Replication of a Novel Tick-borne Bunyavirus through Interacting with Viral Nonstructural Protein NSs. J Biol Chem. 2016, 291(31):16138-49.

115. Chen Yuyan, Frost S, Khushi M, et al. Delayed recruiting of TPD52 to lipid droplets - evidence for a "second wave" of lipid droplet-associated proteins that respond to altered lipid storage induced by Brefeldin A treatment. Sci Rep. 2019, 9(1):9790.

116. Liao HJ, Tsai HF, Wu CS, et al. TRAIL inhibits RANK signaling and suppresses osteoclast activation via inhibiting lipid raft assembly and TRAF6 recruitment. Cell Death Dis. 2019, 10(2):77.

117. Wang Chenran, Haas MA, Yang Fuchun, et al. Autophagic lipid metabolism sustains mTORC1 activity in TSC-deficient neural stem cells. Nat Metab. 2019, 1(11):1127-1140.

118. Olzmann JA, Richter CM, Kopito RR. Spatial regulation of UBXD8 and p97/VCP controls ATGL-mediated lipid droplet turnover. Proc Natl Acad Sci U S A. 2013, 110(4):1345-1350.

119. Zhao YG, Chen Yong, Miao Guangyan, et al. The ER-Localized Transmembrane Protein EPG-3/VMP1 Regulates SERCA Activity to Control ER-Isolation Membrane Contacts for Autophagosome Formation. Mol Cell. 2017, 67(6):974-989.

120. Tábara LC, Escalante R. VMP1 Establishes ER-Microdomains that Regulate Membrane Contact Sites and Autophagy. PLoS One. 2016, 11(11):e0166499.
